# Potential role of microbiome in Chronic Fatigue Syndrome/Myalgic Encephalomyelits (CFS/ME)

**DOI:** 10.1038/s41598-021-86425-6

**Published:** 2021-03-29

**Authors:** Giuseppe Francesco Damiano Lupo, Gabriele Rocchetti, Luigi Lucini, Lorenzo Lorusso, Elena Manara, Matteo Bertelli, Edoardo Puglisi, Enrica Capelli

**Affiliations:** 1 Department for Sustainable Food Process-DiSTAS, Università Cattolica del Sacro Cuore, Via Emilia Parmense 84, 29122 Piacenza, Italy; 2 Laboratory of Immunology and Genetic Analysis, Department of Earth and Environmental Science, University of Pavia, 27100 Pavia, Italy; 3 Centre for Health Technologies, University of Pavia, 27100 Pavia, Italy; 4 ASST Lecco, UOC Neurology and Stroke Unit, Merate, LC Italy; 5 MAGI Euregio, Via Maso della Pieve, 60/A, 39100 Bolzano, Italy

**Keywords:** Clinical microbiology, Microbial ecology, Microbiome, Metabolomics

## Abstract

Chronic Fatigue Syndrome/Myalgic Encephalomyelitis (CFS/ME) is a severe multisystemic disease characterized by immunological abnormalities and dysfunction of energy metabolism. Recent evidences suggest strong correlations between dysbiosis and pathological condition. The present research explored the composition of the intestinal and oral microbiota in CFS/ME patients as compared to healthy controls. The fecal metabolomic profile of a subgroup of CFS/ME patients was also compared with the one of healthy controls. The fecal and salivary bacterial composition in CFS/ME patients was investigated by Illumina sequencing of 16S rRNA gene amplicons. The metabolomic analysis was performed by an UHPLC-MS. The fecal microbiota of CFS/ME patients showed a reduction of *Lachnospiraceae,* particularly *Anaerostipes,* and an increased abundance of genera *Bacteroides* and *Phascolarctobacterium* compared to the non-CFS/ME groups. The oral microbiota of CFS/ME patients showed an increase of *Rothia dentocariosa*. The fecal metabolomic profile of CFS/ME patients revealed high levels of glutamic acid and argininosuccinic acid, together with a decrease of alpha-tocopherol. Our results reveal microbial signatures of dysbiosis in the intestinal microbiota of CFS/ME patients. Further studies are needed to better understand if the microbial composition changes are cause or consequence of the onset of CFS/ME and if they are related to any of the several secondary symptoms.

## Introduction

Chronic Fatigue Syndrome (CFS), also known as Myalgic Encephalomyelitis (ME), is a severe debilitating multisystemic disease that involves nervous^1^, immune^2,3^, endocrine^4^, digestive^5^, and skeletal^6^ systems and is associated with dysfunctions of both energy metabolism and cellular ion transport^7^. The main symptom of CFS/ME is a persistent unexplained fatigue together with other symptoms such as cognitive dysfunctions, unrefreshing sleep, post-exertional malaise, myalgia and joint pain^8^.

CFS/ME occurs with inflammatory symptoms and is characterized by an “abnormal” immune response after physical exertion^9^. Alterations of cell-mediated immunity, with a reduction in Natural Killer cells response and a strong increase in pro-inflammatory cytokines levels have been described in CFS/ME^10,11^. Some features of the syndrome such as the “relapsing–remitting” symptoms, the higher prevalence in the women and a persistent immune activation suggest a similarity with autoimmune conditions. Gastrointestinal symptoms and cognitive impairment were also frequently observed^1^. Recently, several studies pointed to the important role of microbiota in host health and, conversely, its contribution to disease development^12,13^. Changes in the intestinal microbial composition have been detected in metabolic diseases^14,15^, intestinal disorders^16–19^, autoimmune conditions^20–22^, cancer^23^ and in several neurological disorders^24–26^, highlighting a strong correlation between dysbiosis and pathological conditions. Concerning CFS/ME, different studies have shown an altered composition and a reduced biodiversity in patients^27–31^ but until now, a relationship between the bacterial composition and pathogenesis of CFS/ME could not be fully elucidated. A study on CFS/ME patients from Belgium and Norway showed significant differences of the intestinal microbiota composition among CFS/ME patients and the healthy population, with increased levels in CFS/ME patients of genera *Lactonifactor* within *Firmicutes* phylum and *Alistipes* within *Bacteroidetes* phylum, and a reduction in other *Firmicutes* genera^27^. Wang et al.^31^ described in the oral microbiota of CFS/ME patients a higher relative abundance of genera *Leptotrichia*, *Prevotella* and *Fusobacterium*, and a decreased abundance of genera *Haemophilus*, *Veillonella* and *Porphyromonas*. These outcomes obtained by different studies probably reflect differences attributable to genetic backgrounds and to recruiting criteria of, which affect the homogeneity of sample populations. In addition, it must be underlined that the lack of specific diagnostic criteria means that the syndrome can group together subjects sharing the same symptomatology, which can be however triggered by different factors such as viral or bacterial or xenobiotic agents^32^). Furthermore, other factors, i.e., the influence of the living environment of these patients, such as pollution factors and lifestyle habits, cannot be excluded. Among the environmental factors, the eating habits that condition the composition of the gut microbiota could play a role in the triggering and maintenance of symptoms.

In addition to gut microbiota, oral microbiota represents an important element which deserves to be considered. In fact, oral cavity represents the first line of interaction between host and environment, where microbial communities from environment can colonize and interact with the immune system of the host. For instance, it has been shown that bacteria of the oral cavity can affect neurocognition^33^ by stimulating pro inflammatory cytokines. Moreover, the oral microbiome produces exotoxins with pharmacological actions and endotoxins, which are released into the systemic circulation and stimulate the immune system leading to pathological manifestations^34^.

In light of the above considerations, in this study we applied a 16S rRNA high-throughput sequencing approach to analyze the oral and intestinal bacterial composition of ME/CFS in a population of Italian patients. A first-grade population of relatives and a control population outside the families were studied in parallel, in order to identify a possible effect of lifestyle habits and a microbial profile of CFS/ME syndrome. In addition, a metabolomics pilot study on a small number of subjects representative for each population considered was conducted to evaluate the metabolic profile in feces.

## Results

### Characteristics of the study population

A total of 105 subjects: 35 patients and 70 control subjects were enrolled. The healthy control population constituted of two distinct groups: 35 patients’ relatives without CFS/ME and 35 healthy subjects not belonging to the patients’ families (Table 1). Twenty-six CFS/ME patients were females and 9 were males, with a mean age of 46.4 ± 16.1 years and a Body mass Index (BMI) of 23.1 ± 4.4. Twenty-five patients (71%) claimed gastrointestinal disturbances and 11 of them (31%) showed IBS comorbidity. The majority of patients (85%) suffered from post-exertional malaise. Patients’ relatives had a mean age of 54.4 ± 15.9 years and a BMI of 25.5 ± 5. Sixteen relatives were females and 19 males. The control group included 26 females and 9 males, with a mean age of 55.2 ± 18 years and a BMI of 23.5 ± 4.7. Seven patients’ relatives (20%) reported gastrointestinal symptoms, while only two relatives (5%) showed post-exertional malaise. No external controls and patients’ relatives reported IBS diagnosis. All participants were Caucasian, followed an omnivorous diet and belonged to the same geographical area (Northern Italy). No one was a smoker. CFS subjects completed the Chalder Fatigue scale (Chalder et al*.* 1993) and the MOS SF-36 Health Survey (Ware et al*.* 1992).

**Table 1:** Characteristics of study population.

	CFS patients	Relatives	Controls
**Gender**			
Female	26	16	26
Male	9	19	9
**Ethnicity/nationality**	Caucasian/Italian	Caucasian/Italian	Caucasian/Italian
**Geographic area**	Northern Italy	Northern Italy	Northern Italy
**Age**			
Mean ± SD	46.4 ± 16.1	54.4 ± 15,9	55.2 ± 18
Median	49	55	58
**BMI**			
Mean ± SD	23.1 ± 4.4	25.5 ± 5	23.5 ± 4.7
Median	22.6	25.3	22
**Smoke**			
Smoker	–	–	–
No smoker	35 (100%)	35 (100%)	35 (100%)
**Diet**			
Omnivorous	35 (100%)	35 (100%)	35 (100%)
Vegetarian	–	–	–
Vegan	–	–	–
**GI symptoms**			
Yes	25 (71%)	7 (20%)	–
No	10 (29%)	28 (80%)	35 (100%)
**Post-exertional malaise**			
Yes	30 (85%)	2 (5%)	–
No	5 (15%)	33 (95%)	35 (100%)
**IBS co-morbidity**			
Yes	11 (31%)	–	–
No	24 (69%)	35 (100%)	35 (100%)
**Chalder Fatigue Scale**			
Range: 0–33	24.9 ± 5.1	–	–
**SF-36 Health Survey**			
Range (%) 0–100		–	–
Physical functioning	52% ± 2.4	–	–
Role limitations due to physical health	13% ± 3.1	–	–
Role limitations due to emotional problems	69% ± 4.7	–	–
Energy/fatigue	23% ± 1.5	–	–
Emotional well-being	65% ± 1.2	–	–
Social functioning	33% ± 2.2	–	–
Pain	49% ± 2.8	–	–
General health	24% ± 1.2	–	–
Health change	50% ± 2.5	–	–

*BMI *Body mass index, *GI* gastrointestinal, *IBS* Irritable Bowel Syndrome.

### Sequencing data analyses of intestinal and oral microbiota

Illumina sequencing data were pre-processed for the OTU- and taxonomy-based analyses. After the assembly and demultiplexing of the Illumina paired-end sequences, a total of 9,967,562 and 4,774,706 raw sequences was obtained for fecal and salivary samples respectively. After quality trimming and chimera checking, these were reduced to 8,788,426 and 3,508,491 filtered sequences. To avoid biases in diversity estimates due to differences in the number of sequences per sample, rarefaction to a common minimum number of 6100 and 5200 sequences per sample was performed for fecal and salivary samples respectively, which represent the number of sequences of the lowest populated sample. After the rarefaction step, 481,803 high quality sequences for fecal samples and 585,579 high-quality sequences for salivary samples remained for further analyses. A high average Good’s coverage (98.53% ± 0.64%) was obtained for fecal samples, highlighting that most of bacterial diversity in the samples was represented. Conversely, in salivary samples the Good’s coverage index was lower (79.90% ± 1.94%).

Subsequently, the reads were clustered in OTUs (Operational Taxonomic Units) at 97% similarity, without any sub-filtering for rare OTUs, and resulted in a total of 4231 OTUs for fecal samples and 78,000 OTUs for salivary samples, with an average number of 207 and 1235 OTUs per sample respectively.

### Analyses of gut microbiota

The diversity within the intestinal bacterial community in all participants was evaluated determining different α-diversity indices (Table 2). No significant difference in terms of total measured OTUs was recorded among groups, even if the patients’ relatives showed a slightly lower richness. Chao1 and Simpson’s evenness indices resulted slightly increased in CFS/ME patients, but the differences were not statistically significant.

**Table 2:** OTU-based diversity indexes in fecal samples of the experimental groups of subjects.

α-Diversity indices	P (mean)	R (mean)	C (mean)	p-value (ANOVA)
Chao1	453.4 ± 194,7	366.9 ± 155.9	422.0 ± 151.2	0.14
Observed Species	215.6 ± 78,0	189.2 ± 74.0	221.4 ± 60.8	0.25
Simpson	17.7 ± 11,1	15.6 ± 9.0	13.3 ± 7.3	0.31

The table shows the total number of OTUs, (b) Chao index and (c) Simpson’s evenness indexes in CFS patients, patients’ relatives and external control group respectively. Univariate (ANOVA) test is applied in order to assess significant differences between groups (p < 0.05).

*P* CFS/ME patients, *R* patients’ relatives, *C* external control group.

**Figure 1:**
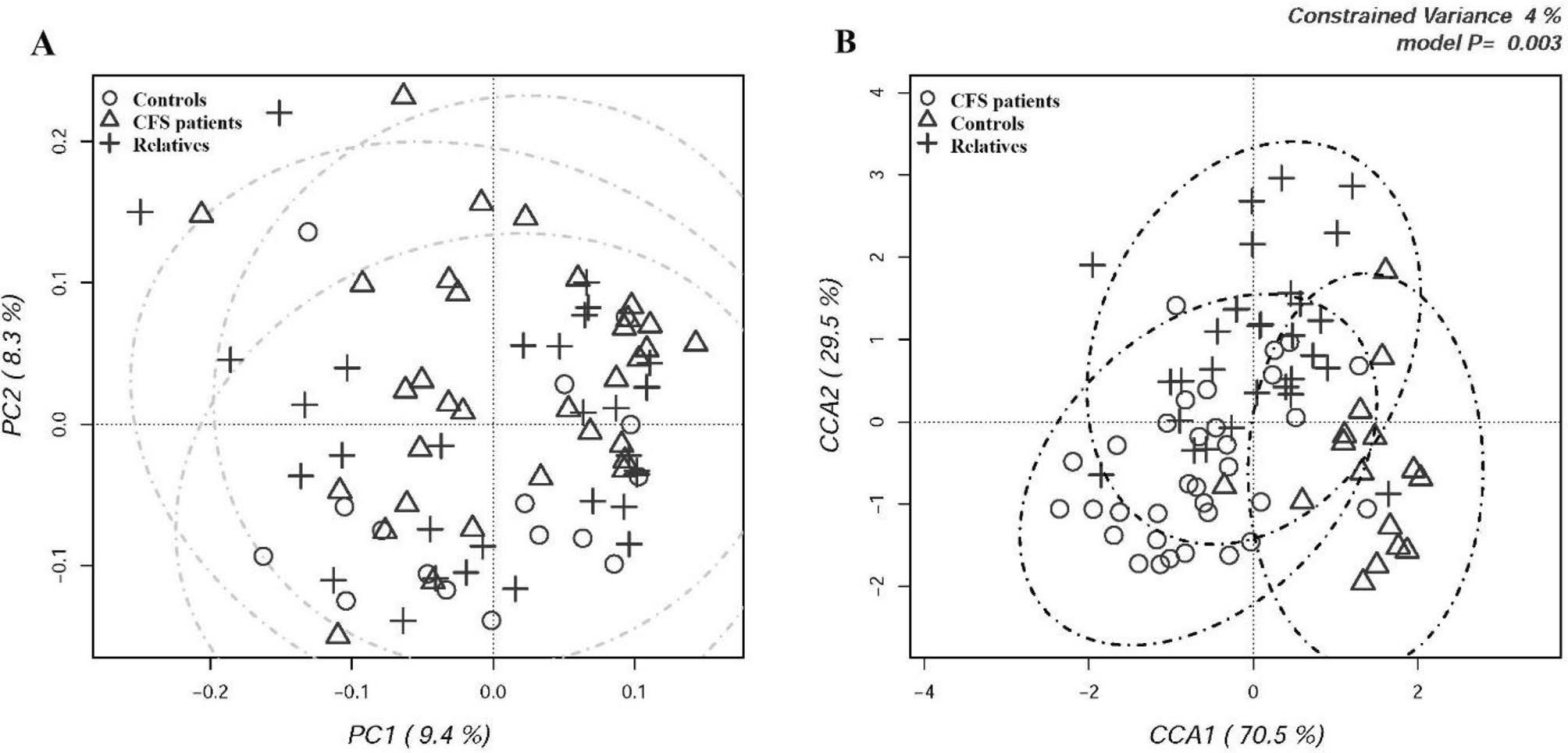
(**A**) Principal component analysis (PCA) and (**B**) Canonical correspondence analysis (CCA) of fecal microbiota compositions in the experimental groups of subjects. PCA and CCA were performed based on abundance of classified OTUs with frequency > 99.9% in CFS patients, patients’ relatives and external healthy controls, respectively. The percentages on each axis indicate the variation in the samples. In CCA (**B**), the plot shows that the disease status is a significant source of variability in bacterial communities, explaining 4% of the variance in fecal microbiota. Individuals are labeled according to the three groups studied.

Multivariate β-diversity analyses were carried out by Principal Component Analysis (PCA, Fig. 1A) and by Canonical Correspondence Analysis (CCA, Fig. 1B) in order to evaluate the dissimilarity between the intestinal microbiota of all participants.

No difference in β-diversity was recorded among groups. According to β-diversity indices, PCA results showed a full overlapping of CFS/ME patients, their relatives and external controls. Microbial diversity was further examined by CCA analysis in order to assess if a classification variable, in this case the healthy state, might influence the relative distribution of OTUs across individuals. Three partially overlapping clusters were recorded (*p* = 0.003), although the model explained only 4% of variance. CFS/ME patients were separated from their relatives and the external control group along the first major axis, while the second axis separated CFS/ME patients and external controls from patients’ relatives.

**Figure 2:**
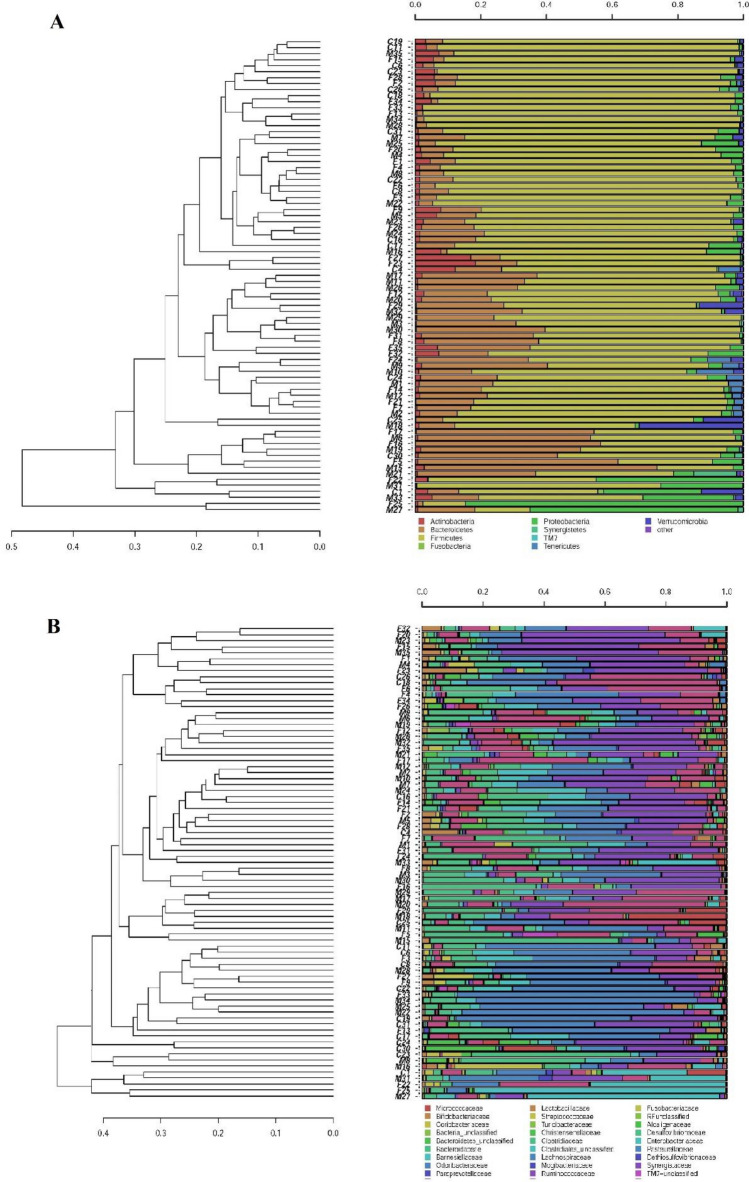
Hierarchical cluster analysis of classified sequences at phylum (**A**) and family (**B**) classification levels. The three experimental groups are indicated as M (CFS patients), F (patients’ relatives), and C (external healthy controls). Bars of different colors indicate the relative percentage of bacterial phyla identified in each fecal sample. Only taxa participating with ≥ 5% in at least one sample are shown, while taxa with lower participations were added to the “other” sequence group. Similar samples were clustered using the average linkage algorithm.

For each sequence, hierarchical clustering analysis was to determine the relative distribution of different bacterial taxa among samples and assess the grouping of individuals performed at the different taxonomic levels. At phylum level, some CFS/ME patients clustered separately because of a reduction in the abundance of *Firmicutes*, mirrored by an increase in the relative abundance of *Bacteroidetes* (Fig. 2A) However, a clear separation of all CFS/ME patients from the other two groups was not observed. When OTUs were classified at the family level, a partial grouping of some CFS/ME patients (M12-M2-M10-M7-M24) was observed, showing a higher relative proportion of *Bacteroidaceae* and a lower abundance of *Lachnospiraceae* (Fig. 2B).

The significant differences between experimental groups in the relative abundances of intestinal bacterial taxa are reported in Table 3. At phylum level, CFS/ME patients, internal and external controls showed that their intestinal bacteria clustered within five main bacterial phyla: *Firmicutes*, *Bacteroidetes*, *Proteobacteria*, *Actinobacteria* and *Verrucomicrobia* (Supplementary Fig. 1). A reduction of *Firmicutes* and a significant increase in *Bacteroidetes* (p = 0.0005) were observed in CFS/ME patients compared to external controls (Supplementary Fig. 2), with a reduction of F/B ratio of about 60% in CFS/ME patients and of 50% in their relatives compared to the external control group. Among the most interesting observed variations, a reduced abundance of *Clostridiales* within the *Firmicutes* phylum was observed in CFS/ME patients (p = 0.023) and a significant increased abundance of *Bacteroidales*, within *Bacteroidetes* phylum, were observed in patients (p = 0.0005) and in their relatives (p = 0.015) when compared to external controls.

**Table 3:** Significant differences in bacterial taxa abundances in fecal samples of the three experimental groups.

Taxon	Relative abundance (%)			Comparisons producing p value < 0.05 (t-test)
	Patients	Relatives	Controls	
**Phylum**				
Bacteroidetes	21.16%	17.40%	10.80%	CFS patients vs controls (0.0105)
**Class**				
**Phylum Bacteroidetes**				
Bacteroidia	20.70%	17.12%	8.63%	CFS patients vs controls (0.0005) Relatives vs controls (0.0157)
**Phylum Firmicutes**				
Clostridia	64.28%	67.79%	76.96%	CFS patients vs controls (0.0239)
Order				
**Phylum: Firmicutes**				
Clostridiales	64.26%	67.77%	76.95%	CFS patients vs controls (0.0238)
**Phylum Bacteroidetes**				
Bacteroidales	20.70%	17.12%	8.63%	CFS patients vs controls (0.0005) Relatives vs controls (0.0157)
Family				
**Phylum: Firmicutes**				
Lachnospiraceae	18.07%	20.56%	30.11%	CFS patients vs controls (0.0345)
**Phylum: Bacteroidetes**				
Bacteroidaceae	9.93%	6.59%	1.27%	CFS patients vs controls (0.0002) relatives vs controls (0.0030)
Barnesiellaceae	1.71%	1.15%	0.27%	CFS patients vs controls (0.0005) Relatives vs controls (0.0089)
**Genus**				
Phylum: Bacteroidetes	Fam: Bacteroidaceae			
Bacteroides	9.93%	6.59%	1.27%	CFS patients vs controls (0.0002) Relatives vs controls (0.0030)
Phylum: Firmicutes	Fam:	Lachnospiraceae		
Anaerostipes	0.18%	0.47%	1.15%	CFS patients vs controls (0.0220) Relatives vs controls (0.0326)
Phylum: Firmicutes	Fam:	Veillonellaceae		
Phascolarctobacterium	1.17%	0.76%	0.15%	CFS patients vs controls (0.0043)
Phylum: Firmicutes	Fam:	Ruminococcaceae		
Ruminococcus	6.60%	11.48%	8.21%	CFS patients vs relatives (0.0194)
**Species**				
Phylum: Bacteroidetes	Fam:	Bacteroidaceae		
Bacteroides uniformis	1.23%	0.91%	0.14%	CFS patients vs controls (0.00005) Relatives vs controls (0.0169)
Bacteroides ovatus	0.98%	1.01%	0.10%	CFS patients vs controls (0.0001) Relatives vs controls (0.0021)
Phylum: Firmicutes	Fam:	Ruminococcaceae		
Ruminococcus bromii	2.25%	4.89%	1.89%	Relatives vs controls (0.0314)

The relative abundances are reported as average abundance values of each group. Student’s t-test and post hoc Bonferroni correction were applied to determine any significant difference in the relative abundances of intestinal bacterial taxa between groups.

At the family level (Supplementary Fig. 3), *Lachnospiraceae* were reduced in CFS/ME patients (p = 0.034) compared to external controls; conversely, *Bacteroidaceae* were about 10 times more abundant in CFS/ME patients (p = 0.0002) and 6 times more abundant in their relatives (p = 0,003) in comparison with external controls; *Barnesiellaceae* resulted significantly increased in both patients (p = 0.0005) and their relatives (p = 0.0089).

At genus level (Supplementary Fig. 4), a significant reduction of *Anaerostipes* was observed in both CFS/ME patients (p = 0.02) and their relatives (p = 0.032) compared to external controls. Differently, *Bacteroides* and *Phascolarctobacterium* were about 10 times more abundant in CFS/ME patients (p = 0.0002 and 0.004, respectively); *Bacteroides* was about 6 times more abundant in patients’ relatives (p = 0.003) and, in the same group, *Ruminococcus* resulted significantly increased (p = 0.0194).

Only 30% of the reads were resolved at species level (Supplementary Fig. 5). Among them, a significant difference was observed for *Bacteroides ovatus* and *Bacteroides uniformis*, which were found to be about 10 times more abundant in both CFS/ME patients (p = 0.0001 and p = 0.00005, respectively) and their relatives (p = 0.0021 and p = 0.0169, respectively) than in external controls. *Ruminococcus bromii* showed a significant increase only in patients’ relatives compared to external controls (p = 0.0314).

### Analyses of salivary microbiota

The α-diversity indices were estimated for each salivary sample. Results showed no differences regarding the total Observed species, the Chao1 and Simpson’s diversity indices among CFS/ME patients, their relatives and the external control group (Table 4).

**Table 4:** OTU-based diversity indexes in salivary samples of the experimental groups of subjects.

α-Diversity indices	P (mean)	R (mean)	C (mean)	p-value (ANOVA)
Chao1	9522.5 ± 1521.5	9793.04 ± 2118.9	9347.0 ± 2217.9	0.65
Observed species	1257.9 ± 97.1	1216.9 ± 91.5	1235.7 ± 139.4	0.37
Simpson	27.02 ± 11.0	27.3 ± 14.4	28.4 ± 15.2	0.91

The table shows the total number of OTUs, (b) Chao index and (c) Simpson’s evenness indexes in CFS patients, patients’ relatives and external control group respectively. Univariate (ANOVA) test is applied in order to assess significant differences between groups (p < 0.05).

*P* CFS/ME patients, *R* patients’ relatives, *C* external control group.

**Figure 3:**
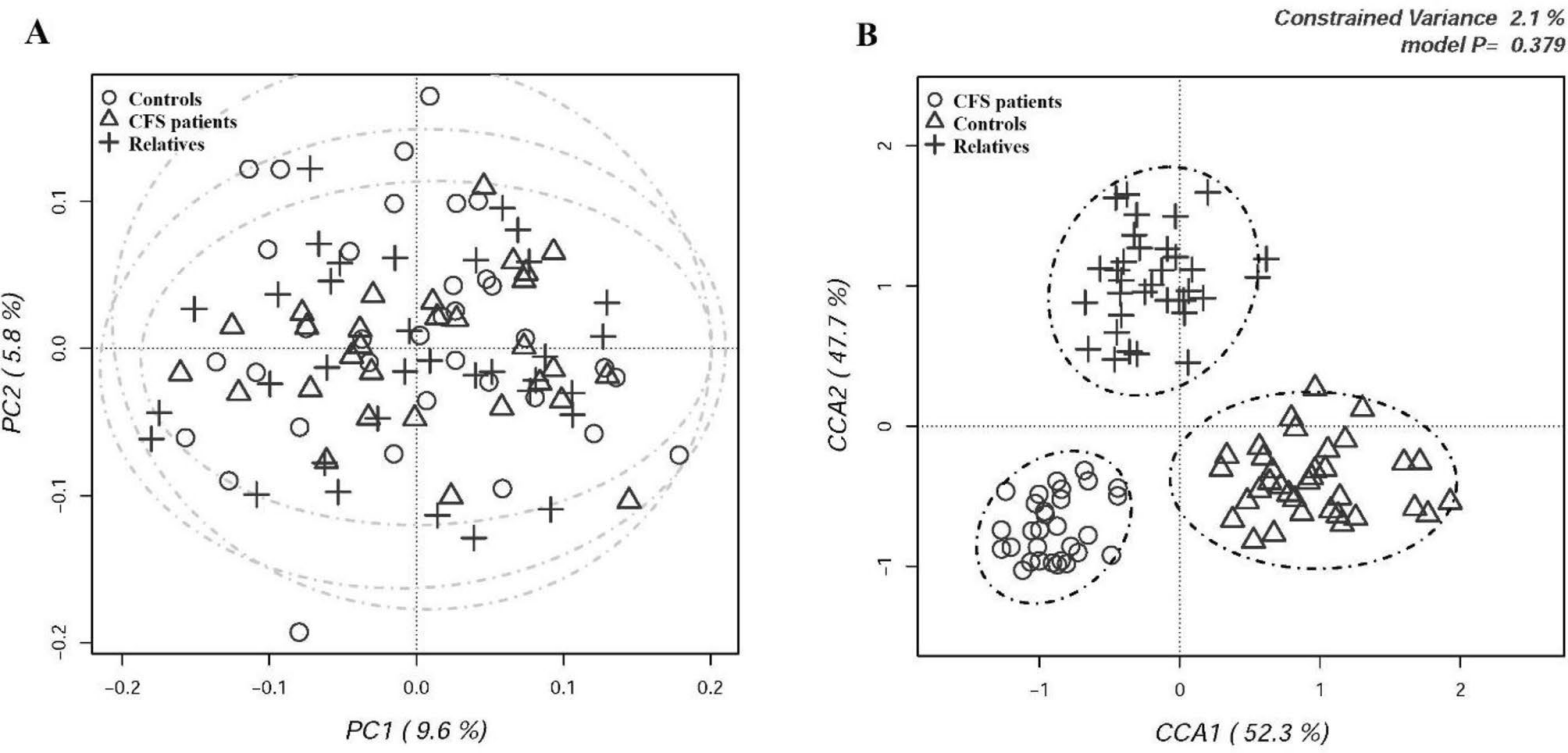
(**A**) Principal component analysis (PCA) and (**B**) Canonical correspondence analysis (CCA) of salivary microbiota compositions in the experimental groups of subjects. PCA and CCA were performed based on abundance of classified OTUs with frequency > 99.9% in CFS patients, patients’ relatives and external healthy controls, respectively. The percentages on each axis indicate the variation in the samples. In CCA, the plot shows that the disease status is not a significant source of variability in bacterial communities, explaining only 2.1% of the variance in salivary microbiota. Individuals are labeled according to the three groups studied.

The distances between the oral microbiome of all participants was assessed by PCA and by CCA on OTUs abundance table in order to evaluate if the microbial community distribution across individuals was affected by the health state. PCA results demonstrated a full overlapping of CFS patients, their relatives and external controls (Fig. 3A). CCA results were illustrated in the Fig. 3B: despite CCA showed a clear discrimination between samples with CFS/ME patients that clustered apart from the other two groups, the model was not significant (p = 0.379) and explained only 2.1% of the total variance.

**Figure 4:**
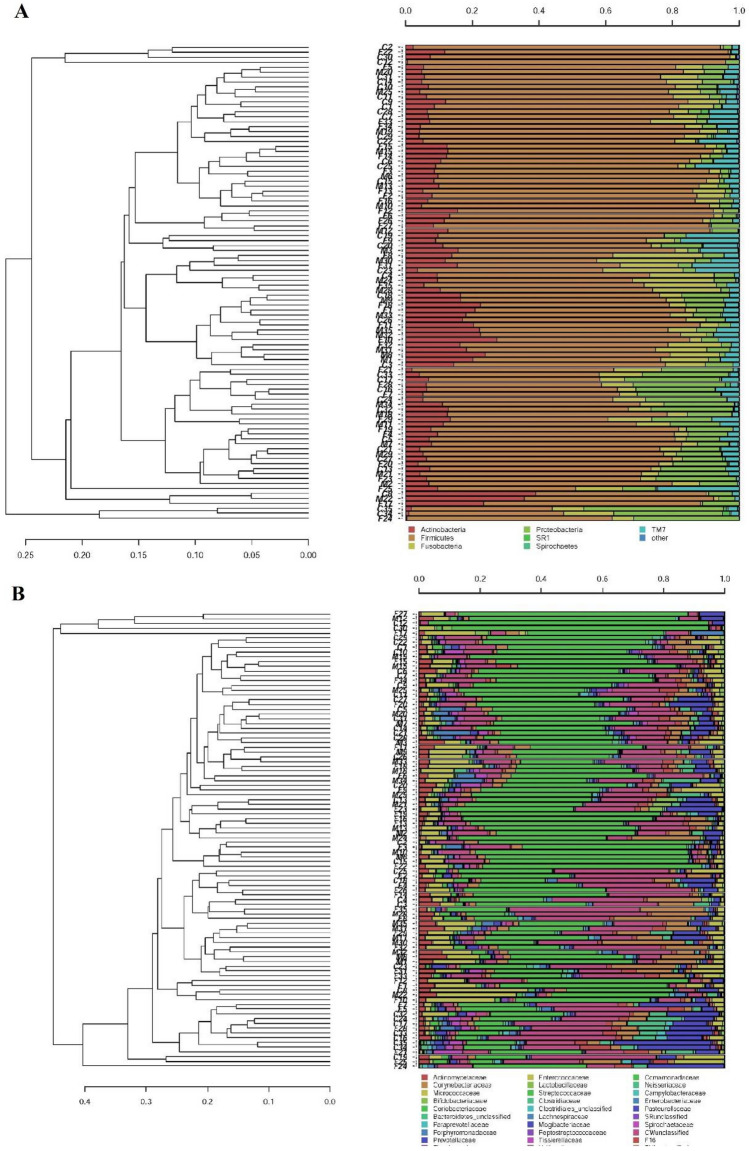
Hierarchical cluster analysis of classified sequences at phylum (**A**) and family (**B**) classification levels. The three experimental groups are indicated as M (CFS patients), F (patients’ relatives), and C (healthy controls). Bars of different colors indicate the relative percentage of bacterial phyla identified in each salivary sample. Only taxa ≥ 5% in at least one sample are shown. Similar samples were clustered using the average linkage algorithm.

When clustering analysis was carried out at phylum and family levels (Fig. 4A,B), no grouping was obtained, indicating that the oral microbiomes of the three experimental groups were similar. The significant differences between the experimental groups in the relative abundances of oral bacterial taxa are reported in Table 5. The salivary bacteria grouped within six main bacterial phyla: *Firmicutes*, *Actinobacteria*, *Proteobacteria*, *Fusobacteria*, *Bacteroidetes* and *TM7* (Supplementary Fig. 6). The predominant phylum *Firmicutes,* did not differed in terms of relative abundances among the three experimental groups. *Actinobacteria* resulted significantly increased in CFS/ME patients (p = 0.01) compared to external controls. The most interesting variation was observed for the order *Actinomycetales*, which resulted significantly higher in CFS/ME patients (p = 0.005) than in the external controls. At family level (Supplementary Fig. 7), the greatest variation was observed for *Micrococcaceae*, significantly more abundant in CFS/ME patients (p = 0.014). Within the *Micrococcaceae* family, the genus *Rothia* was significantly more abundant in CFS/ME patients (p = 0.014) (Supplementary Fig. 8). At species level (Supplementary Fig. 9), the most evident differences were observed for *Rothia mucilaginosa* and *Rothia dentocariosa*, both belonging to *Actinobacteria* phylum, with the latter significantly increased both in CFS/ME patients (p = 0.034) and in their relatives (p = 0.010) compared to external control group.

**Table 5:** Significant differences in bacterial taxa abundances in salivary samples of the three experimental groups.

Taxon	Relative abundance (%)			Comparisons producing p value < 0.05 (t-test)
	Patients	Relatives	Controls	
**Phylum**				
Actinobacteria	12.29%	10.47%	7.92%	CFS patients vs controls (0.014)
**Order**				
Actinomycetales	11.05%	9.03%	6.38%	CFS patients vs controls (0.005)
**Family**				
**Phylum: Actinobacteria**				
Micrococcaceae	7.44%	6.39%	3.98%	CFS patients vs controls (0.014)
**Genus**				
Phylum: Actinobacteria	Fam:	Micrococcaceae		
Rothia	7.44%	6.38%	3.97%	CFS patients vs controls (0.014)
**Species**				
Phylum: Actinobacteria	Fam:	Micrococcaceae		
Rothia dentocariosa	1.03%	1.53%	0.50%	CFS patients vs controls (0.034) Relatives vs controls (0.010)

The relative abundances are reported as average abundance values of each group. Student’s two-tailed t-test and post hoc Bonferroni correction were applied to determine any significant difference in the relative abundances of oral bacterial taxa between groups.

### UHPLC-MS analysis

Based on the results of 16S sequencing analysis of the intestinal microbiota characterizing CFS/ME patients, an untargeted metabolomics study (UHPLC-QTOF) was conducted on the fecal samples of five selected CFS/ME patients (i.e., M12-M2-M10-M7-M24) which were found belonging to a same cluster at family level. This approach was used to evaluate whether the metabolomic profile of CFS/ME patients differed from those of their relatives and controls outside the family. Therefore, five relatives of CFS/ME patients and five external controls were also selected.

**Figure 5:**
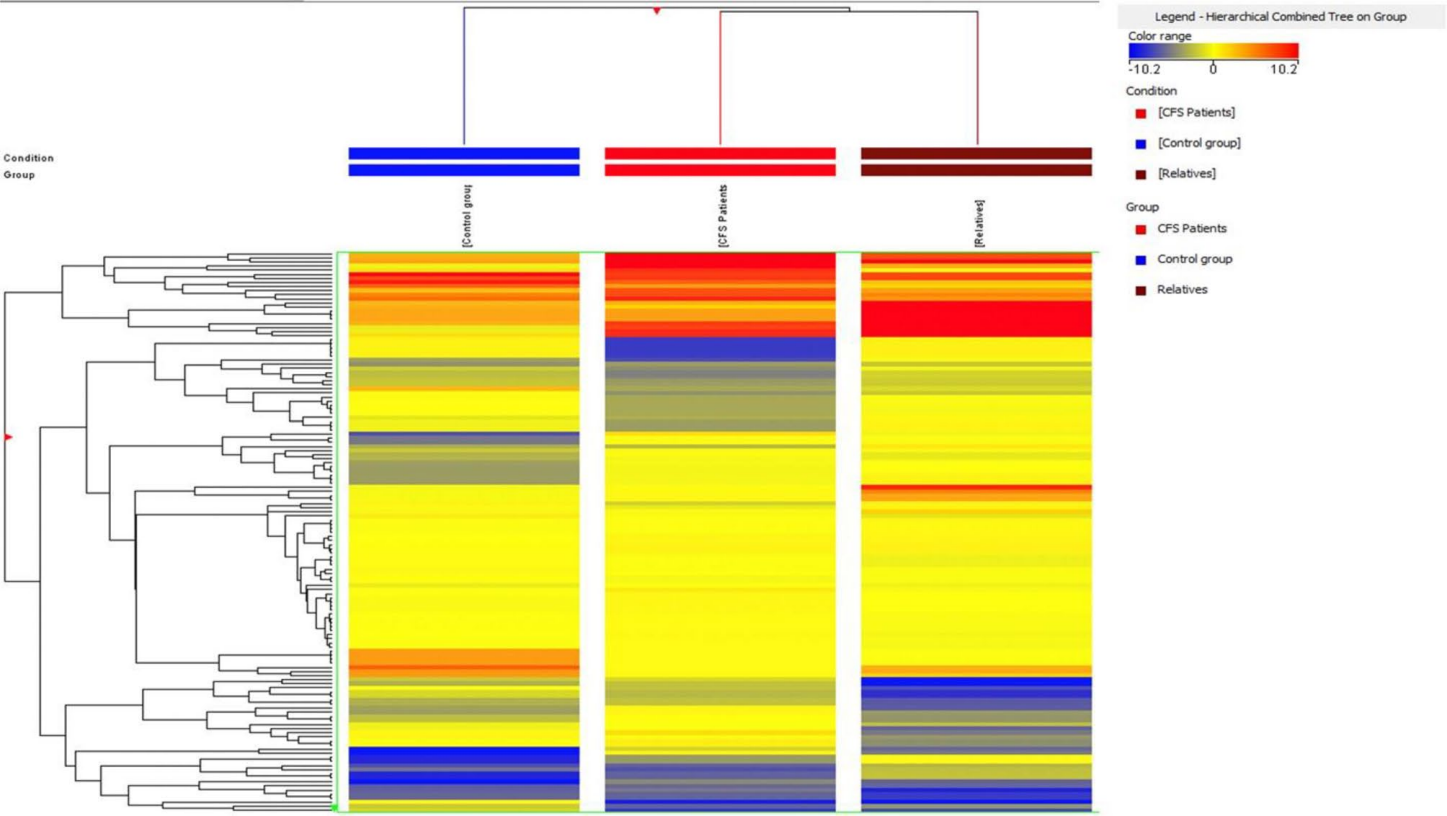
Unsupervised hierarchical cluster analysis (HCA) of identified metabolites in fecal samples of CFS/ME patients, the relatives and the external healthy controls.

The UHPLC-MS analysis was carried out according to an untargeted metabolomics-based approach and resulted in 4848 annotated raw mass features. However, after data deconvolution and identification against the comprehensive Fecal Metabolome database, we obtained 176 annotated compounds, each one reported in Supplementary Information (Supplementary Table 1) together with its composite mass spectrum. Overall, the most represented classes were fatty acyls (35 compounds), followed by amino acids, peptides, and analogues (28 compounds), organooxygen compounds (19 annotations), and polyphenols (12 compounds, mainly benzoic acids). Besides, according to the nature of the matrix analyzed, we found several steroid derivatives. The tandem-MS approach based on QCs confirmed the identity of some metabolites, such as leucine, tyrosine, 2-aminooctanoic acid, alpha-linoleic acid, oleamide, indoleacrylic acid, 1-phenylethylamine, sphingosine, sphinganine, oleanolic acid, nutriacholic acid, and mesobilirubinogen. Thereafter, to represent similarities/dissimilarities in metabolomic profiles between the three different sample groups (i.e., control, CFS/ME patients, and relatives), an unsupervised hierarchical cluster analysis (HCA) was inspected. The output of this analysis is reported in Fig. 5, as the average value of the five biological replicates. The clustering obtained from the heat map (based on Fold-Change distribution) clearly showed the similarities between the fecal metabolome of CFS/ME patients and their relatives, whilst the control group was characterized by a more specific profile. In particular, it was evident that specific groups of metabolites were up- and down-accumulated with a different magnitude when considering the three groups analyzed. Considering the intriguing differences emerging from unsupervised HCA, further statistical approaches based on Volcano plot analysis and supervised PLS and OPLS discriminant analyses, were exploited to find those metabolites able to better discriminate CFS/ME patients and their relatives from the controls. Overall, when considering the comparison "CFS/ME patients *vs* control group" (Supplementary Fig. 10A), the Volcano plot analysis revealed 7 discriminant compounds, that are reported in Table 6 together with their LogFC variation and *p*-value. As can be observed from the Table 6, the significant compounds resulting up accumulated in CFS/ME were glutamic acid (LogFC = 1.93; p < 0.05) and argininosuccinic acid (LogFC = 17.29; p < 0.05), whilst alpha-tocopherol was the metabolite presenting the highest down accumulation trend (LogFC = − 17.39; p < 0.05). The same statistical approach for the comparison "relatives vs control group" (Supplementary Fig. 10B) provided a different list of discriminant markers, that are listed in Table 6. In fact, 8 fecal metabolites were characterized by significant perturbations, with the relatives group characterized by an up accumulation of lithocholyltaurine (LogFC = 18.30; p < 0.05) and eicosapentaenoic acid (LogFC = 19.99; p < 0.05). Interestingly, alpha-tocopherol was found to be a marker of CFS/ME patients and relatives, presenting also a strong down accumulation (LogFC = − 17.29; p < 0.05) for the relatives group.

**Table 6:** Annotated metabolites which significantly differed between patients (CFS), their relatives (REL) compared to the external controls (CTR).

Metabolites	Fold-change (CFS vs CTR)	log2(FC)	p-value adjusted	(−)log10(p)	p[1]	p(corr)[1]	VIP score
l-Glutamic acid	3.8047	1.9278	1.46E−03	2.8348	− 10.274	− 0.81071	3.0293
alpha-Tocopherol	5.86E−06	− 17.38	4.30E−02	1.3667	8.0793	0.66417	2.3586
Argininosuccinic acid	161.230	17.299	4.30E−02	1.3667	− 7.8981	− 0.65018	2.3557
L-Lysine	7.22E−06	− 17.079	4.30E−02	1.3667	7.7921	0.64855	2.327
PC1819Z/1819Z	0.0025782	− 8.5994	1.20E−03	2.9193	7.8345	0.83144	2.3071
8-Hydroxyguanine	0.34112	− 1.5517	4.30E−02	1.3667	7.6075	0.64709	2.3027
3b-Hydroxy-5-cholenoic acid	0.20909	− 2.2578	3.16E−02	1.5004	5.9675	0.70329	1.7651

Metabolites	Fold-change (REL vs CTR)	log2(FC)	p-value ajusted	(−)log10(p)	p[1]	p(corr)[1]	VIP score
Lithocholyltaurine	323.340	18.303	2.01E−03	2.6961	10.92	0.80287	2.8555
8-Hydroxyguanine	0.079125	− 3.6597	2.01E−03	2.6961	− 10.398	− 0.80962	2.6482
Eicosapentaenoic acid	1.042400	19.991	4.24E−02	1.3724	9.0595	0.64775	2.359
alpha-Tocopherol	6.22E−06	− 17.294	4.24E−02	1.3724	− 8.6469	− 0.66472	2.1926
Azelaic acid	4.16E−06	− 17.874	4.24E−02	1.3724	− 8.1545	− 0.62498	2.1851
myo-Inositol 1-phosphate	0.5541	− 0.85177	1.13E−02	1.9474	− 2.6435	− 0.7181	0.6855
Glucose 6-phosphate	0.5541	− 0.85177	1.13E−02	1.9474	− 2.6435	− 0.7181	0.6855
Mannose 6-phosphate	0.5541	− 0.85177	1.13E−02	1.9474	− 2.6435	− 0.7181	0.6855

Metabolites with VIP (Variable Importance in Projection) scores > 1 represented the most contributory variables in group discrimination in the OPLS-DA model. Fold changes were the ratio of the average MS ion intensities (peak areas) in CFS patients, their relatives and external controls. ANOVA was applied to assess statistical significance (p < 0.05). p[1] and p(corr)[1] represented the modeled covariance and the correlation profile in the S-plot, respectively. Variable with higher p[1] in both positive and negative directions have a large impact on the variance between groups, whereas variables with higher p(corr)[1] values have more reliability.

**Figure 6:**
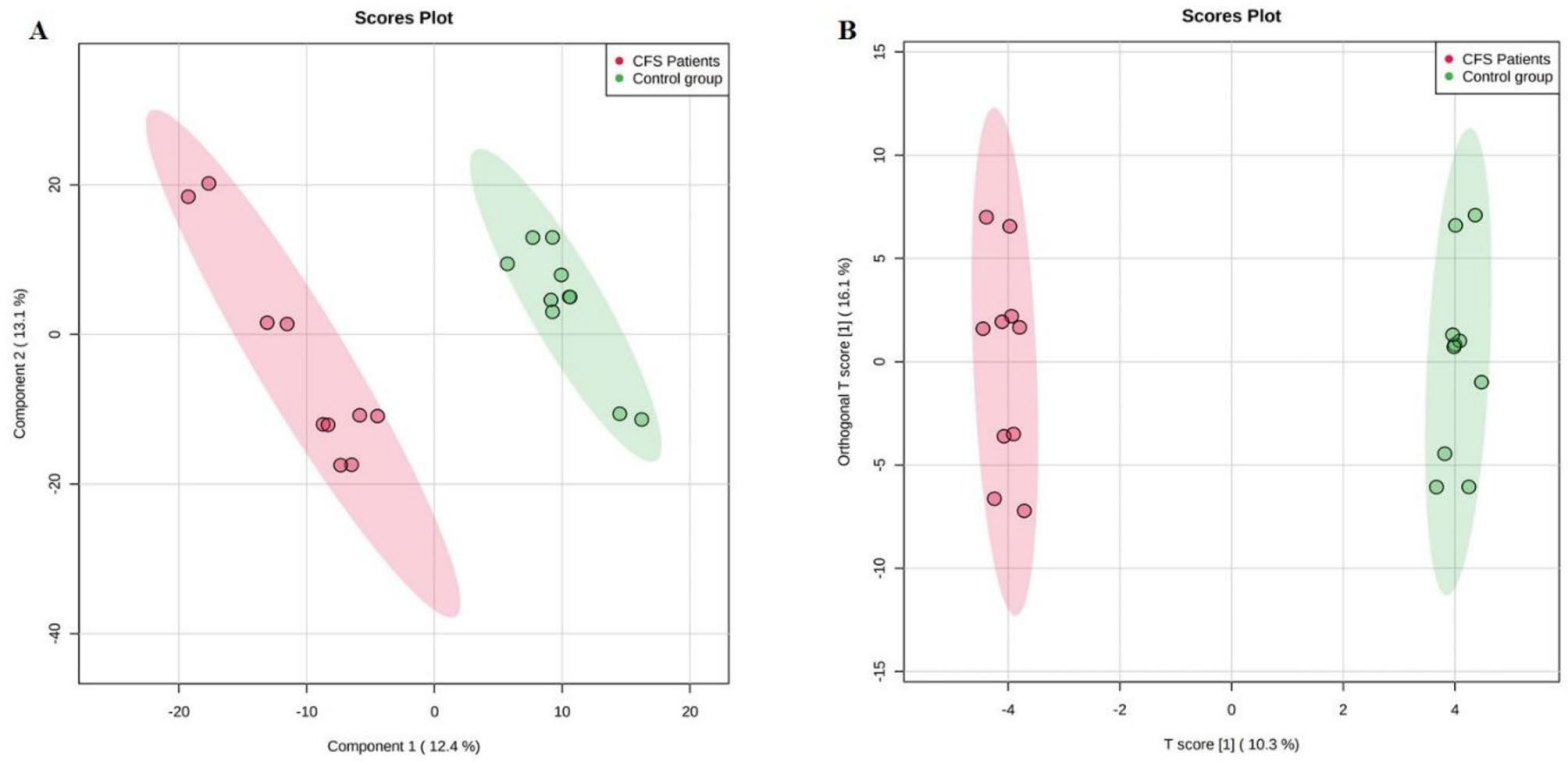
(**A**) PLS-DA and (**B**) OPLS-DA supervised models for the classification of the experimental groups (CFS patients vs controls). In the score plot of OPLS-DA, the x-axis represents the biological component and the y-axis the orthogonal component.

As next step, multivariate supervised methods, namely partial least squares and orthogonal projection to latent structures discriminant analysis (i.e., PLS-DA and OPLS-DA, respectively), have been used to better identify discriminant compounds allowing sample grouping. Using a class membership (typical of supervised models) allows a better separation between classes in the score plot space, mainly because the Y-predictive variability is well separated from the Y-uncorrelated in X (i.e., orthogonal component). The output of both PLS-DA and OPLS-DA score plots resulting from the comparison with the control group for both CFS/ME patients and relatives, is reported in Fig. 6 (A and B, respectively). A clear discrimination was obtained by looking at both biological and technical replicates into the score plot hyperspace, with CFS/ME patients and relatives presenting distinct fecal metabolomic signatures compared to control. Besides, the VIP approach following PLS-DA modelling for the comparison "CFS/ME patients vs control group", showed 49 metabolites possessing a VIP score > 1 (Supplementary Table 2), with the highest values recorded for glutamic acid (VIP score = 3.03), followed by alpha-tocopherol (VIP score = 2.36), and argininosuccinic acid (VIP score = 2.35), thus corroborating the results of the Volcano plot analysis. The significant features of the OPLS-DA modelling were then extrapolated by inspecting the S-plot. This approach combines the contribution (covariance) with the effect and reliability (correlation) for the model variables with respect to model component scores. As can be observed from the Supplementary Table 3), glutamic acid and argininosuccinic acid were highlighted again as two of the most discriminant metabolites, able to clearly discriminate CFS/ME patients from the control group. Similar information was outlined when comparing the relatives with the control group. In fact, as can be observed from the Supplementary Fig. 11 (Supplementary Information), both PLS-DA and OPLS-DA supervised modelling discriminated the two-sample population. In this case, a higher number of discriminant metabolites was outlined by VIP selection method (i.e., 55 compounds), with the 22.4% of VIP markers shared with the CFS/ME patients. In fact, as revealed by a Venn diagram (Supplementary Fig. 12), 30 metabolites were found to characterized exclusively the CFS/ME fecal metabolome. The OPLS-DA models built were cross-validated and permutation testing excluded overfitting. Besides, each supervised OPLS-DA model was characterized by more than acceptable goodness parameters, being R^2^Y(cum) > 0.80 and Q^2^(cum) > 0.54.

## Discussion

The present study highlighted significant variations in both the intestinal and oral bacteria composition between CFS/ME patients, their relatives and external controls, as shown by changes in the relative abundances of several bacterial taxa. It is also worth noting that in most cases the patients’ relatives showed intermediate prevalence values. β-diversity analyses showed a reduced inter-individual variability between CFS/ME patients and healthy subjects for both the salivary and fecal microbiota.

A reduction of *Firmicutes* and, on the contrary, a significant increase of *Bacteroidetes* in CFS/ME patients in comparison with the non-CFS/ME groups was shown by the fecal microbiota analyses. The prevalence of these phyla showed intermediate values in patients’ relatives, suggesting an environmental influence.

These results agree with those of previous studies, reporting that the intestinal microbiome in CFS/ME patients is characterized by a decreased of *Firmicutes* and an increasing of *Bacteroidetes*^27–29^. A similar pattern was observed also in the fecal microbiome of patients with Alzheimer’s disease^35^. The microbiome is indeed known to play an important role in gut-brain communication through the gut-brain axis^36^, synthesizing a large number of chemical mediators, which can reach distant sites, such as the brain, exerting positive or negative effects on the host health^37^.

We also found a decrease of the *Firmicutes*/*Bacteroidetes* (F/B) ratio both in CFS/ME patients and in their relatives: this shift has been already previously described in various autoimmune conditions, such as Chron’s disease^18^, Systemic Lupus Erythematous^21^, and diabetes type 2^38^. In our study, the reduction of *Firmicutes* along with the increased proportion of *Bacteroidetes* observed in both CFS/ME patients and their relatives was mainly ascribed to members of *Clostridiales* in CFS/ME subjects and of *Bacteroidales* in their relatives. Among *Clostridiales*, several families declined, with *Lachnospiraceae* showing the greatest decrease. *Lachnospiraceae* are abundant in the gastrointestinal tract of humans^39,40^. All members belonging to this family are strictly anaerobic and spore-forming bacteria, and several species are important butyrate producers^41^. The capability of producing butyric acid and its important role for both microbial and host epithelial cell growth, link the members of this family to both obesity^42,43^ and protection from colon cancer in humans^44^. A low abundance of *Lachnospiraceae* has been observed in pediatric patients with ulcerative colitis and Chron’s disease^19^, both autoimmune conditions. Moreover, members belonging to this family were also underrepresented in the gut of adult IBD patients^45^. The decrease of *Lachnospiraceae* involved the genera *Roseburia*, *Coprococcus*, *Lachnospira* and *Anaerostipes*. All these are dominant genera in the healthy human gut and most of them are butyrate producers; butyric acid is a SCFA representing an important source of energy for other microbes and for the host’s enterocytes and contributes to the maintenance of the intestinal barrier, so protecting gut epithelium against inflammation^46^. In particular, we observed a significant reduction of *Anaerostipes* both in CFS patients and in their relatives. These bacteria are able to convert lactate into butyrate and were found to be reduced in smoking IBD patients^47^. The decreased abundance of butyrate-producing bacteria observed in CFS/ME patients, could result in the alteration of the intestinal barrier integrity with a consequent increased susceptibility to inflammation.

Other interesting modifications were observed in *Bacteroidales*: a significant increase of *Bacteroidaceae* and *Barnesiellaceae* and particularly, of *Bacteroides*, in both CFS/ME patients and their relatives. *Bacteroides* is the predominant genus in the lower human intestinal tract. These bacteria play a fundamental role in the processing of complex molecules in the host intestine, particularly in carbohydrate fermentation. This activity results in the production of SCFA, mainly propionate and acetate, used by the host as energy source^48^. The oral administration of *Bacteroides uniformis* CECT 7771, a butyrate-producing bacterium of the human gut, was found to ameliorates metabolic and immunological dysfunction in mice with high-fat-diet induced obesity^49^.

Although *Bacteroides* species are normally commensals in the human gut flora, some of them are opportunistic pathogens that have been found in most infections of the peritoneal cavity, particularly appendicitis through abscess formation^50^, and can cause anaerobic bacteremia^51^. *Bacteroides* possess virulence factors involved in the adherence to tissues, protection from the host’s immune response and consequent destruction of tissues. The lipopolysachcarides (LPS), also known as endotoxin, can induce a high inflammatory response when enter into the bloodstream^52^ and are able to affect intestinal homeostasis by either changing the gut permeability or triggering immune reaction inside the intestinal mucosa. High levels of LPS, were detected in the bloodstream of subjects with CFS/ME^29^.

An over-representation of proteins, largely derived from *Bacteroides* species, which allow these opportunistic pathogens to break the intestinal barrier and invade the mucosa^53^ was detected in individuals affected by Chron’s disease. Some *Bacteriodes* species are indeed able to damage the intestinal barrier by means of their virulence factor and may compromise the permeability of the intestinal barrier, resulting in a “*leaky gut*”, and to promote bacterial translocation in the bloodstream, causing an abnormal systemic inflammatory response.

In CFS patients we recorded also a large increase of *Phascolarctobacterium*, an acetate/propionate-producer, abundant in the human gut. The increased abundance of this bacterium could be related to dietary habits as reported in rats fed with fat rich showing a higher amount of SCFAs producers, including *Phascolarctobacterium faecium*^54^.

The analysis of salivary microbiota revealed a significantly increase of *Actinobacteria* in CFS patients with a higher prevalence of *Actinomycetales*. Within this order we observed, only in CFS/ME patients, a significant increase of *Micrococcaceae,* particularly *Rothia sp*. *Rothia* is a Gram-positive and aerobic bacterial genus inhabiting the mouth and respiratory tract. In our study we identified two pathogenic species belonging to this genus: *Rothia dentocariosa* and *Rothia mucilaginosa*, but the increase was significant only for the latter. This species is implicated in periodontal disease, which may represent the first step in the infection of other body sites including brain^55^ and coronary arteries^56^. *R. dentocariosa* is involved in the infection of lung, tonsils, peritoneum and brain^57–59^.

The fecal metabolomic profile of CFS/ME patients, as assessed by UHPLC-QTOF mass spectrometry analysis, proved to be different from that of both relatives and external controls. In fact, intriguing discriminant markers emerged from the different multivariate statistical approaches following data deconvolution and identification. Overall, the unsupervised clustering (HCA) provided a first interesting information, i.e., that the fecal metabolomic profile of CFS/ME samples were more similar to that of their relatives, compared to control group. The similarities observed from the heat map based on fold-change values (Fig. 5) revealed that some metabolites were strictly common to both CFS/ME patients and their relatives, likely due to similar diets and lifestyles. In this regard, several phenolic metabolites resulting from a catabolic activity into the gut on parent compounds (i.e., higher molecular weight polyphenols, such as flavonoids) have been detected, likely resulting from the consumption of fruits and vegetables. Overall, common abundant compounds were found to be bile acid derivatives, likely produced by microbial enzymes from colonic environment and urobilogens. These compounds, according to the Fecal Metabolome database, have been listed under the name steroids and steroid derivatives. In this regard, the enzyme bilirubin-UDP-glucuronyltransferase (bilirubin-UGT) is known to process bilirubin in hepatocytes to produce the more water-soluble version of the molecule known as bilirubin diglucuronide. The bilirubin diglucuronide is transferred rapidly across the canalicular membrane into the bile canaliculi where it is then excreted as bile into the large intestine. The bilirubin is further reduced by gut microbiota of the large intestine to form a colorless product known as urobilinogen. This latter, when remaining in the colon environment, can either be reduced to stercobilinogen and then oxidized to stercobilin, or directly reduced to stercobilin. In our experimental conditions, we detected both mesobilirubinogen and stercobilin. Interestingly, another abundant class of fecal metabolites detected (35 compounds) was that of fatty acyls. Among fatty acyls, the 3-methylthiopropionic acid (one of the metabolites of methionine) was found to be the most abundant and common metabolite (Supplementary Table 1). Besides, in our experimental conditions, some alkaloids were annotated, such as trigonelline, widely described as a biomarker of the consumption of coffee, legumes and soy products. Afterwards, by using Volcano plot analysis and multivariate statistical approaches (such as PLS-DA and OPLS-DA) the most significant compounds allowing the discrimination of CFS/ME patients from the control group have been detected. As general consideration, few significant compounds resulted from the Volcano plot analysis (Table 6), while the VIP selection method following PLS-DA supervised modelling revealed a higher number of significant features (i.e., 49; Supplementary Table 2). However, it was interesting to notice that some metabolites were common significant markers for each statistical tool used. Among these, a strong degree of discrimination was promoted by glutamic acid and argininosuccinic acid, showing positive accumulation value in CFS/ME patients compared to control group. In this regard, some recent works^60^ outlined the involvement of glutamic acid in the so-called microbiota-gut brain axis, acting as a neurotransmitter and/or neuromodulator in this bidirectional axis. Glutamic acid found in the gut principally derives from dietary proteins and from free glutamate characterizing certain food additives^61^. However, a fraction of the free glutamate in the lumen originates from bacterial synthesis, mainly by lactic acid bacteria^62^. This aminoacid is also involved as an excitatory neurotransmitter in both central nervous system and in the enteric nervous system, where the amino acid is synthesized by neurons and glial cells^60^. According to literature, the modulation of glutamatergic receptors along the microbiota–gut–brain axis may influence several physiologic responses. Besides, the alterations of the glutamatergic transmission may be a factor in the development of pathologies involving derangement of this bidirectional axis. This aspect is extremely important, considering that a high accumulation of glutamate is known to promote the excitotoxicity, which can damage or destroy neurons. Also, due to the low blood flow problems in chronic fatigue syndrome, central nervous system ischemia increases glutamate levels and contributes to cellular death. Normally glutamate is balanced by the inhibitory neurotransmitter GABA, so an excess of this compound leads to various overloud phenomena. Therefore, as highlighted in scientific literature, the possible initiating factors to CFS could be due to oxidative stress/weak or compromised antioxidant capacity^63^, mitochondrial dysfunction^64^ or glutamate/GABA imbalances^65,66^. Taken together, our findings outline the need to perform further studies (using a wider sample population and considering also the diet habits of each subject) focusing on the possible involvement of changes in glutamatergic signaling along the microbiota–gut–brain axis in the development of CFS/ME.

Regarding the other discriminant metabolites outlined by multivariate statistics for the comparison CFS/ME patients *vs* control group, a strong up-accumulation of argininosuccinic acid was shown (Table 6). According to literature^67^, CFS was reported to affect mainly the intermediate metabolite concentrations in the tricarboxylic acid (TCA) and urea cycles. In particular, Yamano et al.^67^ proposed the ratio of ornithine/citrulline (detected in plasma of CFS/ME individuals) as marker of the CFS status. However, in our experimental conditions, no statistical differences were detected for the different intermediates of urea cycle, except for argininosuccinate. This latter is a very important intermediate, in fact, the enzyme argininosuccinate lyase (ASL) cleaves argininosuccinate into arginine and fumarate, as part of the nitric oxide (NO)-citrulline cycle that regulates NO production in multiple tissues^68^. Interestingly, the ASL deficiency has been reported to reduce the levels of arginine, thus determining also increased ammonia and reduced nitric oxide levels^69^. In this regard, nitric oxide (NO) is involved in crucial processes in the central nervous system, including neurotransmission, neuronal differentiation and migration^69^. Overall, it was hypothesized that a NO-dependent reduction in inhibitory activity of the central nervous system and consequent central sensitization accounts for chronic widespread pain in CFS/ME patients^70^. Therefore, the up-accumulation of argininosuccinate in CFS/ME patients could be related to a possible alteration of NO-citrulline cycle, although no clinical evidence has been deeply investigated yet and further studies are mandatory. Finally, another interesting and significant marker detected was alpha tocopherol (vitamin E). This metabolite was down accumulated in both CFS/ME patients and their relatives, compared to control group (Table 6). Overall, the oxidative stress has been previously described as involved in the CFS/ME syndrome^71,72^. In this regard, serum alpha-tocopherol concentrations have been found significantly lower in the CFS/ME patients as compared with the control subjects, thus suggesting increased oxidative stress in the former.

The untargeted metabolomic-based analysis allowed us to hypothesize and confirm different metabolomic alterations, as induced by CFS/ME, likely correlated to NO-citrulline cycles, glutamine cycle, and oxidative stress. However, as reported by Castro-Marrero et al.^73^, to date, no firm conclusions can be drawn regarding possible treatments and management of CFS/ME, because the few existing randomized controlled trials have been small-scale, with a high risk of bias, and have used different case definitions. Therefore, further studies based on rigorous experimental designs and comprehensive data analyses appear to be mandatory, focusing on several and inclusive parameters, such as clinical presentation, patient characteristics, case criteria, and degree of disability^73^.

## Conclusions

The present study reports alterations in the composition of both the fecal and salivary microbiota of CFS/ME patients, with more marked differences observed in the gut. We have confirmed the results of previous studies^27–29^, and provided new information in support of the autoimmune hypothesis for CFS condition^74–77^. The intestinal microbial profile we recorded in CFS/ME patients is indeed consistent with the reported for other autoimmune conditions, such as Crohn’s disease^18^, Ulcerative Colitis^19^ and Systemic Lupus Erythematous^21^.

The significant increase or decrease of several bacterial taxa observed in CFS/ME patients compared to external controls, indicates the presence of a modified microbiome in both saliva and gut of CFS/ME patients. The same type of modifications observed also in their relatives, suggests the involvement of environmental or genetic-related factors. The metabolomic analysis revealed some differences in the fecal metabolic profiles of CFS/ME patients suggesting the possible involvement of changes in glutamatergic signaling along the microbiota–gut–brain axis in the development of CFS/ME. These results must be confirmed in a larger cohort and may lead to a better understanding of the relationship between metabolic changes and CFS/ME-related immunological and cognitive dysfunctions. However, further studies are needed to better understand whether the alteration of the microbiota is a cause or a consequence of the onset of CFS/ME and if the changes in the microbial composition are related to any of the several secondary symptoms. If our results will be confirmed, the differences detected in the microbial and metabolic profiles of CFS/ME patients may be used as markers in addition to diagnostic criteria for a more accurate recognition of the syndrome and for the development of new therapeutic strategies.

## Materials and methods

### Participant recruitment and sample collection

A total of 105 volunteers were enrolled for this study: 35 CFS patients, 35 patients’ relatives without CFS living with patients, and 35 healthy controls. The recruitment was performed according to the following inclusion criteria: CFS diagnosis according to Fukuda’s criteria^78^, age between 18 to 80 years and signature of informed consent.

All participants who had used antibiotics, cortisone and non-steroidal anti-inflammatory drugs, inhibitors of proton pump inhibitors and probiotic drugs in the two months before the recruitment were excluded.

Healthy control subjects and patients’ relatives who had a previous history of diseases associated with chronic fatigue, bacterial and viral infections, cancer, chronic coronary diseases and allergies were also excluded.

Only the relatives who lived with the patients and shared similar dietary habits with them were enrolled. Age, sex and body mass index were matched in healthy controls.

Twenty-five patients reported gastrointestinal symptoms and showed post-exertional malaise. Moreover, eleven patients also had Irritable Bowel Syndrome (IBS) diagnoses. Neither controls nor patients’ relatives reported this syndrome.

The recruitment and sample collection were carried out at the clinic of Laboratory Magi EUREGIO s.c.s of Bozen, thanks to the collaboration of the Italian Association of CFS patients—AMCFS Onlus. Fecal and salivary samples were collected in sterile tubes by the participants themselves and delivered to appointed personnel for storage at − 80 °C within one hour. The study was performed in accordance with the Declaration of Helsinki and was approved by the Ethics Committee of Bozen province, Italy (nr. 65-2019, 16/10/2019). Written informed consent was obtained from all participants.

### Metagenomic analyses

#### DNA extraction

Fecal and salivary samples of all participants were processed using QIAmp DNA Stool Mini kit and the QIAmp DNA Blood Mini kit (Qiagen, Hilden, Germany) respectively, in accordance with the manufacturer instructions. Total genomic DNA was extracted from 200 mg of fecal samples and from 1 mL of salivary samples. The DNA integrity was checked by agarose gel electrophoresis, while DNA concentration was measured by Qubit HS dsDNA fluorescence assay (Life Technologies, Carlsbad, CA, USA).

#### 16S rRNA gene amplification and Illumina sequencing

The PCR amplification of the V3-V4 regions of 16S rRNA was conducted using the indexed primers 343 F (5′-TACGGRAGGCAGCAG-3′) and 802 R (5′-TACNVGGGTWTCTAATCC-3′), as described in Spigni et al. (2018).

PCR reactions were performed in 25 μL containing 12.5 μL of Phusion Flash High-Fidelity Master Mix (Thermo Fisher Scientific, Inc., Waltham, MA, USA), 0.5 μM of each primer, 1 ng of DNA template and PCR nuclease-free water. To analyze several amplicon samples simultaneously in the same sequencing run, a multiplexing strategy was performed. An extension of nine nucleic acids was added to the 5′ end of the forward primer, with the first seven bases acting as a tag, to identify each sample unequivocally, and the other two bases acting as a linker.

The two step-PCR method described in Berry et al.^79^ was adopted, with a first PCR step of 25 cycles using untagged primers, and a second PCR step of 8 cycles with tagged primers and 1 μL of first step products used as template. The PCR thermal profile was the same in the two steps: 30 s of denaturation at 94 °C, 30 s of primer annealing at 50 °C and 30 s of primer elongation at 72 °C, followed by a final elongation step of 10 min at 72 °C.

The final PCR products were verified by electrophoresis on 1% agarose gel and the concentrations of amplicons were measured by Qubit HS dsDNA fluorescence assay (Life Technologies, Carlsbad, CA, USA).

Equimolar concentrations of amplicons (35 ng/µL for fecal samples and 30 ng/µL for salivary samples) were pooled and then purified with the SPRI (Solid Phase Reverse Immobilization) method using the Agencourt AMPure XP kit (Beckman Coulter, Italy, Milano). The purified pool was sent to Fasteris Company (Geneva, Switzerland) for the amplicon library preparation and for paired-end sequencing (2 bp × 300 bp) on MiSeq Illumina platform (Illumina Inc, San Diego, CA), operating with V3 chemistry.

#### Dataset preparation and data analysis

Illumina sequencing data were pre-processed for OTU- and taxonomy-based analyses. Raw paired reads were assembled with the “pandaseq” script^80^, allowing at least 30 bp of overlap between the read pairs and a maximum of two mismatches. Sequences were demultiplexed and quality-controlled with the fastx-toolkit, according to sample indexes (http://hannonlab.cshl.edu/fastx_toolkit/).

Sequences with large homopolymers (≥ 10), sequences that did not align within the targeted V3–V4 region, chimeric sequences^81^, and sequences that were not classified as bacterial after alignment against the Mothur version of the RDP training data set were removed with Mothur v.1.39.5^82^. Mothur and R^83^ were used to analyze the resulting high-quality sequences following both the OTU- and taxonomy-based approach. For the OTU approach, sequences were first aligned against the SILVA reference database release 138 for bacteria^84^ using the NAST algorithm and a kmer approach^85,86^, and then clustered in OTUs (Operational Taxonomic Units) with 97% similarity using the average linkage algorithm. OTUs were classified at taxonomical levels by alignment against the Greengenes database^87^.

Based on OTU matrixes, the α and β diversity indices were calculated using Mothur and R software^83^. α-diversity is used to measure the diversity within a sample. Different metrics were applied to calculate α-diversity indices: Observed species, Chao1 and inverse Simpson. β-diversity is used to estimate the diversity between samples and provides a measure of the distance between each sample pair. Bray–Curtis metric was applied to calculate the indices of dissimilarity.

The good coverage estimate was calculated to evaluate the percentage of diversity captured by sequencing. The most abundant OTUs identified were confirmed by BLAST (Basic Local Alignment Search) searches against the RDP database.

Statistical analyses were carried out on OTU matrixes by using Mothur and R software^83^ and included hierarchical clustering analyses with the average linkage algorithm at different taxonomic levels, principal component analysis (PCA) and canonical correspondence analysis (CCA). ANOVA, Student’s two-tailed t-test and post hoc Bonferroni correction were applied to test for any significant difference in the relative abundances of oral and intestinal bacterial taxa between groups. A p-value threshold of 0.05 was used.

Sequence data were submitted to the National Centre for Biotechnology Information Sequence Read Archive (BioProjects PRJNA702086 for the intestinal 16S dataset and PRJNA702047 for the salivary 16 s dataset).

### Metabolomic analyses

#### Sample preparation and untargeted metabolomic analysis

The extraction of the compounds was performed considering 1 g of fecal material and using 10 mL of aqueous methanol (80% v/v) and 0.1 mL of formic acid. After an overnight incubation at − 18 °C, the samples were centrifuged, and 1 mL of supernatant was filtered using a 0.22 μm cellulose syringe filters and transferred into glass vials for the metabolomic analysis. Also, quality control (QC) samples were done by mixing an aliquot (40 μL) from each sample.

The untargeted profiling of the faecal samples was done using a 1290 UHPLC system coupled with a G6550 quadrupole-time-of-flight (QTOF) mass spectrometer (Agilent Technologies, Santa Clara, CA, United States), using an electrospray ionization system (JetStream dual). The comprehensive description of the optimized instrumental conditions (for both chromatography and mass spectrometry) can be found in previous works^88,89^. The injection volume was 6 μL, considering a Full-Scan mode in the range 100–1200 *m/z*, and working in positive ionization mode (ESI+). The sequence injection was randomized, and QC samples allowed to check both system stability and data quality. In this regard, QCs were injected at the beginning of the sequence and every 8 sample injections, setting a data-dependent MS/MS mode based on the selection of 12 precursor ions per cycle (1 Hz, 50–1200 *m/z*, active exclusion after 2 spectra), with typical collision energies of 10, 20 and 40 eV. The injection sequence was randomized to avoid any possible time-dependent changes during UHPLC-QTOF analysis. Each set of experimental samples was preceded by a blank control (extraction procedure without sample).

The raw mass features from UHPLC-ESI/QTOF were processed as widely detailed in our previous works^88,89^, using the Profinder software (version B.07, from Agilent Technologies) to annotate molecular features following mass and retention time alignment. Thereafter, a 'find-by-formula' algorithm coupled with the isotopic profile of each mass feature was used to annotate the compounds against the comprehensive “Human Fecal Metabolome" database, containing specific information about many small molecule metabolites found in human feces^90^. This approach allowed us to reach a Level 2 of confidence in annotation, as set out by the Metabolomics Standard Initiative (Sumner et al., 2007). Also, a further confirmation step for identification purposes was done elaboration the annotations from the QCs in the software MS-DIAL 4.20^91^. In this regard, the available MS/MS experimental spectra built in the software (e.g., MoNA) and the in-silico fragmentation approach based on MS-FINDER (i.e., using the information reported on Human Metabolome and Human Fecal Metabolome databases)^92^ were used.

#### Multivariate statistical analysis of untargeted metabolomics data

The metabolomics data were elaborated in the software Mass Profiler Professional (from Agilent Technologies) following the annotation step. The abundance of each metabolite detected was normalized at the 75th percentile and then an unsupervised Hierarchical Cluster Analysis (HCA) was used to reveal similarities when considering the three groups under investigations (i.e., control, CFS patients, and relatives). The metabolomic dataset was then loaded into MetaboAnalyst software^93^ for the univariate and multivariate statistical analyses. The Fold-Change (FC) (cut-off > 1.2) and ANOVA analyses (p-value < 0.05, followed by False Discovery Rate-adjustment) were combined into Volcano plot, to inspect for each possible comparison the significantly differential metabolites. Then, partial least squares (PLS) and orthogonal projections to latent structures (OPLS) discriminant analyses (DA) were used as supervised methods in order to maximize the separation between the observed groups. Besides, variable importance in projections in the PLS-DA model (VIP; cut-off > 1) together with significant features from S-plot (combining the covariance and correlation loading profiles with respect to OPLS-DA model component scores) were inspected to obtain discriminant metabolites. The OPLS-DA model was then cross-validated using an ANOVA analysis, (p < 0.01) and permutation testing (N = 200) was then inspected to exclude overfitting, by monitoring also the goodness-of-fit and prediction ability of the supervised models (i.e., R^2^Y and Q^2^Y, respectively).

## Supplementary Information

Supplementary Information 1.

Supplementary Information 2.

## References

[1] Chen R (2008). Chronic fatigue syndrome and the central nervous system. J. Int. Med. Res..

[2] Fletcher MA (2010). Biomarkers in chronic fatigue syndrome: Evaluation of natural killer cell function and dipeptyl peptidase IV. PLoS ONE.

[3] Lorusso L (2009). Immunological aspects of chronic fatigue syndrome. Autoimmun. Rev..

[4] Cleare AJ (2003). The neuroendocrinology of chronic fatigue syndrome. Endocr. Rev..

[5] Lakhan SE, Kirchgessner A (2010). Gut inflammation in chronic fatigue syndrome. Nutr. Metab..

[6] Jones DEJ, Hollingsworth KG, Taylor R, Blamire AM, Newton JL (2009). Abnormalities in Ph handling by peripheral muscle and potential regulation by the autonomic nervous system in chronic fatigue syndrome. J. Int. Med..

[7] Myhill S, Booth NE, McLaren-Howard J (2009). Chronic fatigue syndrome and mitochondrial dysfunction. Int. J. Clin. Exp. Med..

[8] IoM (2015). Beyond Myalgic Encephalomyelitis/Chronic Fatigue Syndrome: Redefining an Illness.

[9] Nijs J (2014). Altered immune response to exercise in patients with chronic fatigue syndrome/myalgic encephalomyelitis: A systematic literature review. Exerc. Immunol. Rev..

[10] Blundell S, Ray KK, Buckland M, White PD (2015). Chronic fatigue syndrome and circulating cytokines: A systematic review. Brain Behav. Immun..

[11] Hornig M (2015). Distinct plasma immune signatures in ME/CFS are present early in the course of illness. Sci. Adv..

[12] Robinson CJ, Bohannan BJM, Young VB (2010). From structure to function: The ecology of host-associated microbial communities. Microbiol. Mol. Biol. Rev..

[13] Bassis C, Young V, Schmidt T (2013). Methods for characterizing microbial communities associated with the human body. Hum. Microbiota..

[14] Qin J (2012). A metagenome-wide association study of gut microbiota in type 2 diabetes. Nature.

[15] Ridaura VK (2013). Gut microbiota from twins discordant for obesity modulate metabolism in mice. Science.

[16] Labus JS (2017). Differences in gut microbial composition correlate with regional brain volumes in irritable bowel syndrome. Microbiome..

[17] Tap J (2017). Identification of an intestinal microbiota signature associated with severity of Irritable Bowel Syndrome. Gastroenterology.

[18] Manichanh C (2006). Reduced diversity of faecal microbiota in Crohn's disease revealed by a metagenomic approach. Gut.

[19] Maukonen J (2015). Altered fecal microbiota in paediatric inflammatory bowel disease. J. Crohns Colitis..

[20] Marasco G (2016). Gut microbiota and celiac disease. Dig. Dis. Sci..

[21] Hevia A (2014). Intestinal dysbiosis associated with systemic lupus erythematosus. MBio.

[22] Patrone V (2017). Gut microbiota profile in systemic sclerosis patients with and without clinical evidence of gastrointestinal involvement. Sci. Rep..

[23] Wu S (2009). A human colonic commensal promotes colon tumorigenesis via activation of T helper type 17 T cell responses. Nat. Med..

[24] Scheperjans F (2015). Gut microbiota are related to Parkinson’s disease and clinical phenotype. Mov. Disord..

[25] Zhuang ZQ (2018). Gut microbiota is altered in patients with Alzheimer's Disease. J. Alzheimers Dis..

[26] Finegold SM (2010). Pyrosequencing study of fecal microflora of autistic and control children. Anaerobe.

[27] Fremont M, Coomans D, Massart S, De Meirleir K (2013). High-throughput 16s rRNA gene sequencing reveals alterations of intestinal microbiota in myalgic encephalomyelitis/chronic fatigue syndrome patients. Anaerobe.

[28] Shukla SK (2015). Changes in gut and plasma microbiome following exercise challenge in myalgic encephalomyelitis/chronic fatigue syndrome (ME/CFS). PLoS ONE.

[29] Giloteaux L (2016). Reduced diversity and altered composition of the gut microbiome in individuals with myalgic encephalomyelitis/chronic fatigue syndrome. Microbiome..

[30] Nagy-Szakal D (2017). Fecal metagenomic profiles in subgroups of patients with myalgic encephalomyelitis/chronic fatigue syndrome. Microbiome..

[31] Wang T (2018). Chronic fatigue syndrome patients have alterations in their oral microbiome composition and function. PLoS ONE.

[32] Arseneau L., Ko G., Elgez A., Romero L. Environmental exposures as a potential underlying factor in chronic fatigue syndrome; a case report. An environmental medicine perspective on a complex syndrome; could toxic exposures be the cause? *Med. Res. Arch*. (2017) 10.18103/mra.v5i12.1573.

[33] Ranjan R., Abhinay A., Mishra M. Can oral microbial infections be a risk factor for neurodegeneration? A review of the literature. *Neurol India*. **66**, 344–51. (2018) https://www.neurologyindia.com/text.asp?2018/66/2/344/227315. 10.4103/0028-3886.227315 29547153

[34] Olsen I, Singhrao SK (2015). Can oral infection be a risk factor for Alzheimer's disease?. J. Oral Microbiol..

[35] Vogt NM (2017). Gut microbiome alterations in Alzheimer’s disease. Sci. Rep..

[36] Zhul X (2017). Microbiota–gut–brain axis and the central nervous system. Oncotarget.

[37] Clarke G (2014). Minireview: Gut microbiota: The neglected endocrine organ. Mol. Endocrinol..

[38] Larsen N (2010). Gut microbiota in human adults with type 2 diabetes differs from non-diabetic adults. PLoS ONE.

[39] Gosalbes MJ (2011). Metatranscriptomic approach to analyze the functional human gut microbiota. PLoS ONE.

[40] Rinninella E (2019). What is the healthy gut microbiota composition? A changing ecosystem across age, environment, diet, and diseases. Microorganisms..

[41] Duncan SH, Hold GL, Barcenilla A, Stewart CS, Flint HJ (2002). *Roseburia intestinalis* sp. Nov., a novel saccharolytic, butyrate-producing bacterium from human faeces. Int. J. Syst. Evol. Microbiol..

[42] Duncan SH (2008). Human colonic microbiota associated with diet, obesity and weight loss. Int. J. Obes. (Lond)..

[43] Turnbaugh PJ, Bäckhed F, Fulton L, Gordon JI (2008). Diet-induced obesity is linked to marked but reversible alterations in the mouse distal gut microbiome. Cell Host Microbe..

[44] Mandal M, Olson DJ, Sharma T, Vadlamudi RK, Kumar R (2001). Butyric acid induces apoptosis by up-regulating Bax expression via stimulation of the c-Jun N-terminal kinase/activation protein-1 pathway in human colon cancer cells. Gastroenterology.

[45] Frank DN (2007). Molecular-phylogenetic characterization of microbial community imbalances in human inflammatory bowel diseases. Proc. Natl. Acad. Sci. U. S. A..

[46] Venegas DP (2019). Short chain fatty acids (SCFAs)-mediated gut epithelial and immune regulation and its relevance for inflammatory bowel diseases. Front. Immunol..

[47] Morgan XC (2012). Dysfunction of the intestinal microbiome in inflammatory bowel disease and treatment. Genome Biol..

[48] Wexler HM (2007). Bacteroides: The good, the bad, and the nitty-gritty. Clin. Microbiol. Rev..

[49] Cano PG, Santacruz A, Moya Á, Sanz Y (2012). Bacteroides uniformis CECT 7771 ameliorates metabolic and immunological dysfunction in mice with high-fat-diet induced obesity. PLoS ONE.

[50] Bennion RS (1990). The bacteriology of gangrenous and perforated appendicitis-revisited. Ann. Surg..

[51] Merchan C (2016). Multidrug-resistant *Bacteroides fragilis* bacteremia in a US resident: An emerging challenge. Case Rep. Infect. Dis..

[52] Zhang R (2009). Circulating endotoxin and systemic immune activation in sporadic amyotrophic lateral sclerosis (sALS). J. Neuroimmunol..

[53] Juste C (2014). Bacterial protein signals are associated with Crohn’s disease. Gut.

[54] Lecomte V (2015). Changes in gut microbiota in rats fed a high fat diet correlate with obesity-associated metabolic parameters. PLoS ONE.

[55] Singhrao SK, Harding A, Poole S, Kesavalu L, Crean SJ (2015). *Porphyromonas gingivalis* periodontal infection and its putative links with Alzheimer’s disease. Mediat. Inflamm..

[56] Mougeot LC (2017). *Porphyromonas gingivalis* is the most abundant species detected in coronary and femoral arteries. J. Oral Microbiol..

[57] Ohashi M (2005). Severe acute tonsillitis caused by Rothia dentocariosa in a healthy child. Pediatr. Infect. Dis. J..

[58] Morris SK, Nag S, Suh KN, Evans GA (2004). Recurrent chronic ambulatory peritoneal dialysis-associated infection due to rothia dentocariosa. Can. J. Infect. Dis. Med. Microbiol..

[59] Ricaurte JC (2001). Rothia dentocariosa endocarditis complicated by multiple intracranial hemorrhages. S. Med. J..

[60] Baj A (2019). Glutamatergic signaling along the microbiota–gut–brain axis. Int. J. Mol. Sci..

[61] Tomé D (2018). The roles of dietary glutamate in the intestine. Ann. Nutr. Metab..

[62] Mazzoli R, Pessione E (2016). The neuro-endocrinological role of microbial glutamate and GABA signaling. Front. Microbiol..

[63] Armstrong CW, McGregor NR, Lewis DP, Butt HL, Gooley PR (2015). Metabolic profiling reveals anomalous energy metabolism and oxidative stress pathways in chronic fatigue syndrome patients. Metabolomics.

[64] Tomas C, Brown AE, Newton JL, Elson JL (2019). Mitochondrial complex activity in permeabilised cells of chronic fatigue syndrome patients using two cell types. PeerJ.

[65] Smith AK, Fang H, Whistler T, Unger ER, Rajeevan MS (2011). Convergent genomic studies identify association of GRIK2 and NPAS2 with chronic fatigue syndrome. Neuropsychobiology.

[66] Glassford JAG (2017). The neuroinflammatory etiopathology of myalgic encephalomyelitis/chronic fatigue syndrome (ME/CFS). Front. Physiol..

[67] Yamano E (2016). Index markers of chronic fatigue syndrome with dysfunction of TCA and urea cycles. Sci. Rep..

[68] Erez A (2011). Requirement of argininosuccinate lyase for systemic nitric oxide production. Nat. Med..

[69] Baruteau J (2017). Expanding the phenotype in argininosuccinic aciduria: Need for new therapies. J. Inherit. Metab. Dis..

[70] Nijs J, Van de Velde B, De Meirleir K (2004). Pain in patients with chronic fatigue syndrome: Does nitric oxide trigger central sensitisation?. Med. Hypotheses.

[71] Miwa K, Fujita M (2010). Fluctuation of serum vitamin E (α-tocopherol) concentrations during exacerbation and remission phases in patients with chronic fatigue syndrome. Heart Vessels.

[72] Miwa K, Masatoshi FM (2009). Increased oxidative stress suggested by low serum vitamin E concentrations in patients with chronic fatigue syndrome. Int. J. Cardiol..

[73] Castro-Marrero J, Sáez-Francàs N, Santillo D, Alegre J (2017). Treatment and management of chronic fatigue syndrome/myalgic encephalomyelitis: All roads lead to Rome. Br. J. Pharmacol..

[74] Patarca R (2001). Cytokines and chronic fatigue syndrome. Ann. N. Y. Acad. Sci..

[75] Skowera A (2002). Antinuclear autoantibodies (ANA) in Gulf War-related illness and chronic fatigue syndrome (CFS) patients. Clin. Exp. Immunol..

[76] Fluge Ø (2011). Benefit from B-lymphocyte depletion using the anti-CD20 antibody rituximab in chronic fatigue syndrome. A double-blind and placebo-controlled study. PLoS ONE.

[77] Sotzny F (2018). Myalgic encephalomyelitis/chronic fatigue syndrome—evidence for an autoimmune disease. Autoimmun. Rev..

[78] Fukuda K (1994). The chronic fatigue syndrome: A comprehensive approach to its definition and study. International Chronic Fatigue Syndrome Study Group. Ann. Intern. Med..

[79] Berry D, Mahfoudh KB, Wagner M, Loy A (2011). Barcoded primers used in multiplex amplicon pyrosequencing bias amplification. Appl. Environ. Microbiol..

[80] Masella AP, Bartram AK, Truszkowski JM, Brown DG, Neufeld JD (2012). PANDAseq: Paired-end assembler for illumina sequences. BMC Bioinform..

[81] Edgar RC, Haas BJ, Clemente JC, Quince C, Knight R (2011). UCHIME improves sensitivity and speed of chimera detection. Bioinformatics.

[82] Schloss PD (2009). Introducing mothur: Open-source, platform-independent, community-supported software for describing and comparing microbial communities. Appl. Environ. Microbiol..

[83] R Core Team. R: A language and environment for statistical computing. R foundation for Statistical Computing. Vienna, Austria (2020). https://www.R-project.org.

[84] Pruesse E (2007). SILVA: A comprehensive online resource for quality checked and aligned ribosomal RNA sequence data compatible with ARB. Nucleic Acids Res..

[85] De Santis TZ (2006). NAST: A multiple sequence alignment server for comparative analysis of 16S rRNA genes. Nucleic Acids Res..

[86] Schloss PD (2010). The effects of alignment quality, distance calculation method, sequence filtering, and region on the analysis of 16S rRNA gene-based studies. PLoS Comput. Biol..

[87] McDonald D (2011). An improved Greengenes taxonomy with explicit ranks for ecological and evolutionary analyses of bacteria and archaea. ISME J..

[88] Senizza A, Rocchetti G, Callegari ML, Lucini L, Morelli L (2020). Linoleic acid induces metabolic stress in the intestinal microorganism Bifidobacterium breve DSM 20213. Sci. Rep..

[89] Rocchetti G (2019). In vitro large intestine fermentation of gluten-free rice cookies containing alfalfa seed (*Medicago sativa* L.) flour: A combined metagenomic/metabolomic approach. Food Res. Int..

[90] Karu N (2018). A review on human fecal metabolomics: Methods, applications and the human fecal metabolome database. Anal. Chim Acta..

[91] Tsugawa H (2015). MS-DIAL: Data-independent MS/MS deconvolution for comprehensive metabolome analysis. Nat. Methods.

[92] Tsugawa H (2016). Hydrogen rearrangement rules: Computational MS/MS fragmentation and structure elucidation using MS-FINDER software. Anal. Chem..

[93] Pang Z, Chong J, Li S, Xia J (2020). MetaboAnalystR 3.0: Toward an optimized workflow for global metabolomics. Metabolites.

