# Supplementary material for: Potential role of microbiome in Chronic Fatigue Syndrome/Myalgic Encephalomyelits (CFS/ME)
Source: Sci Rep. 2021 Mar 29;11:7043. doi: 10.1038/s41598-021-86425-6 (PMC8007739; doi:10.1038/s41598-021-86425-6)
Supplement: Supplementary file 2 — Supplementary Information 2. [file 41598_2021_86425_MOESM2_ESM.pdf]

Potential role of microbiome in Chronic Fatigue Syndrome/Myalgic Encephalomyelits (CFS/ME)

Giuseppe Francesco Damiano Lupo[123], Gabriele Rocchetti[1], Luigi Lucini[1], Lorenzo Lorusso[4], Elena Manara[5], Matteo Bertelli[5], Edoardo Puglisi[1]*, Enrica Capelli[2]*

SUPPLEMENTARY MATERIAL

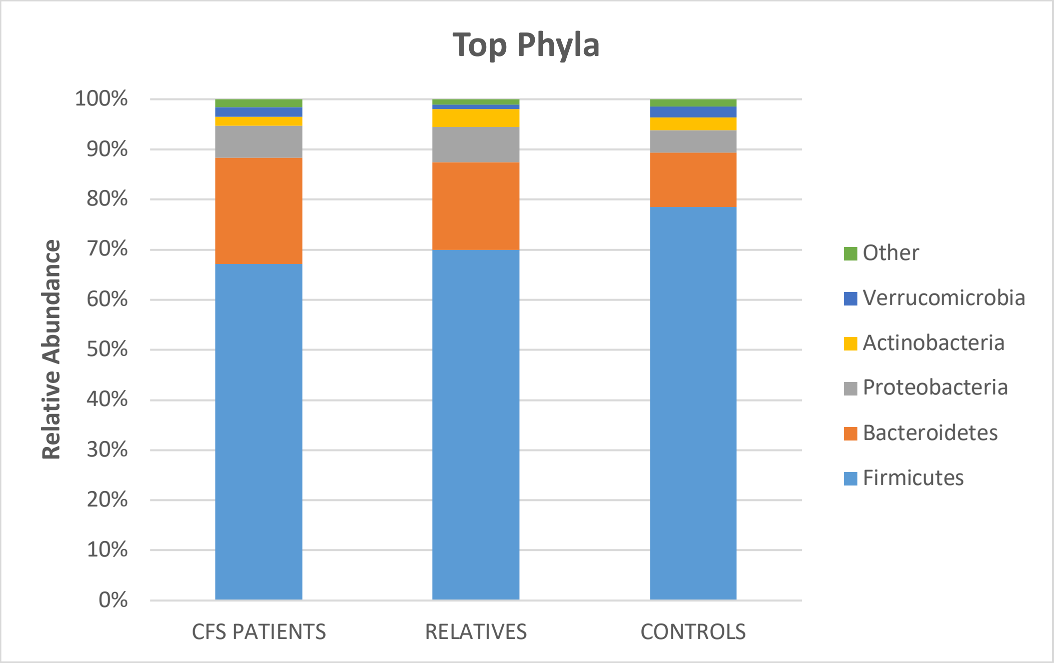

Figure 1. Compositions of the intestinal microbiota from CFS patients, their relatives and controls at phylum level. Only taxa ≥1% in at least one sample are shown, while taxa with lower participations were added to the "other" sequence group.

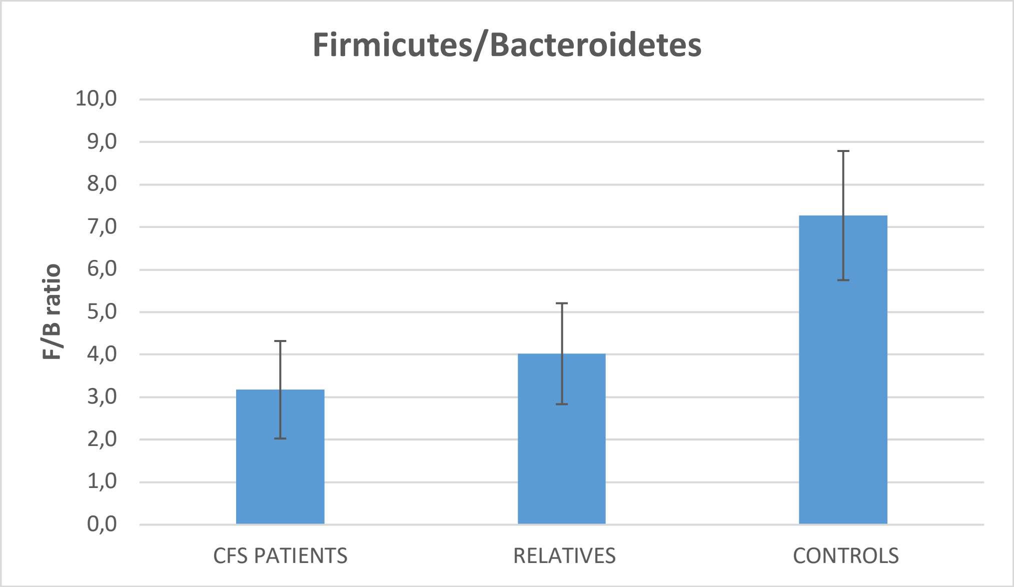

Figure 2. Firmicutes/Bacteroidetes (F/B) ratio in fecal samples across the three experimental groups. Comparison of F/B ratio between CFS patients, their relatives and external controls.

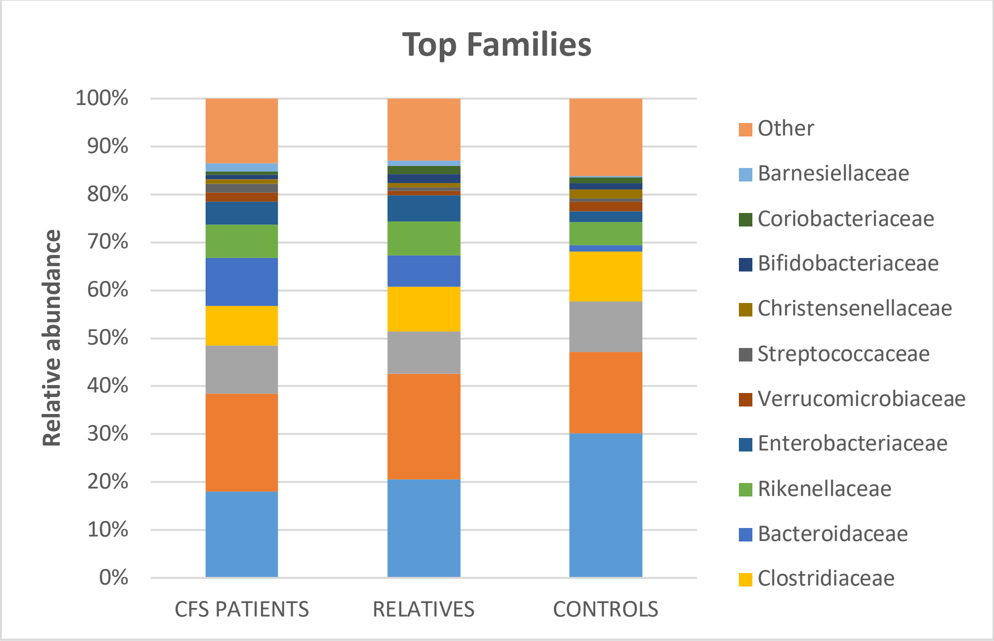

Figure 3. Compositions of the intestinal microbiota from CFS patients, relatives and controls at family level. Only taxa ≥1% in at least one sample are shown, while taxa with lower participations were added to the "other" sequence group.

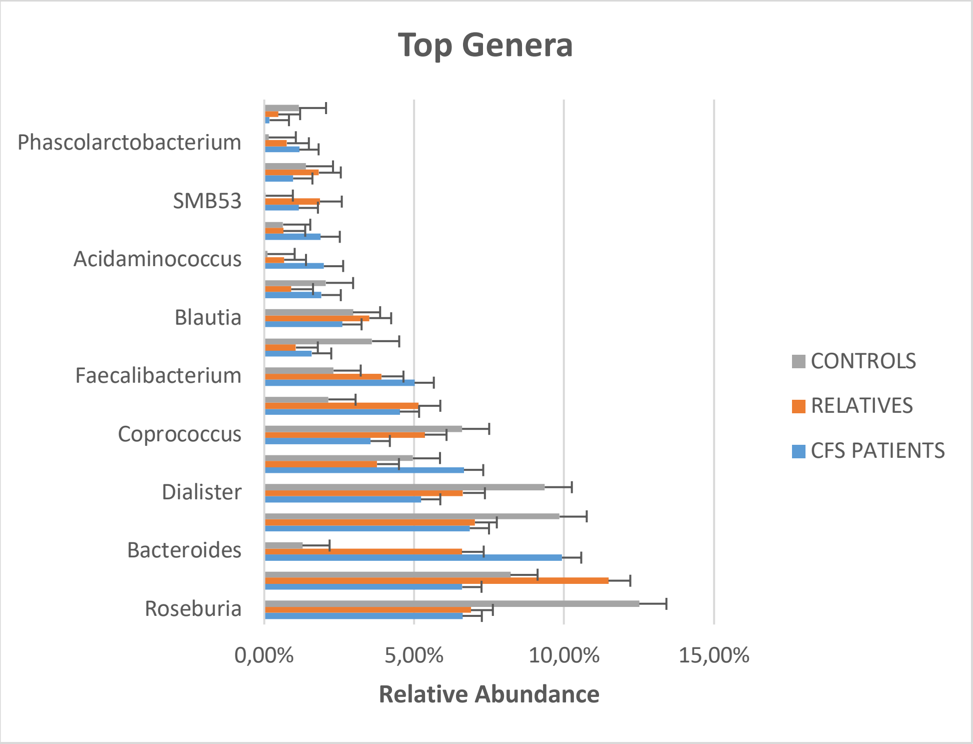

Figure 4. Compositions of the intestinal microbiota from CFS patients, relatives and controls at genus level. Only taxa ≥1% in at least one sample are shown.

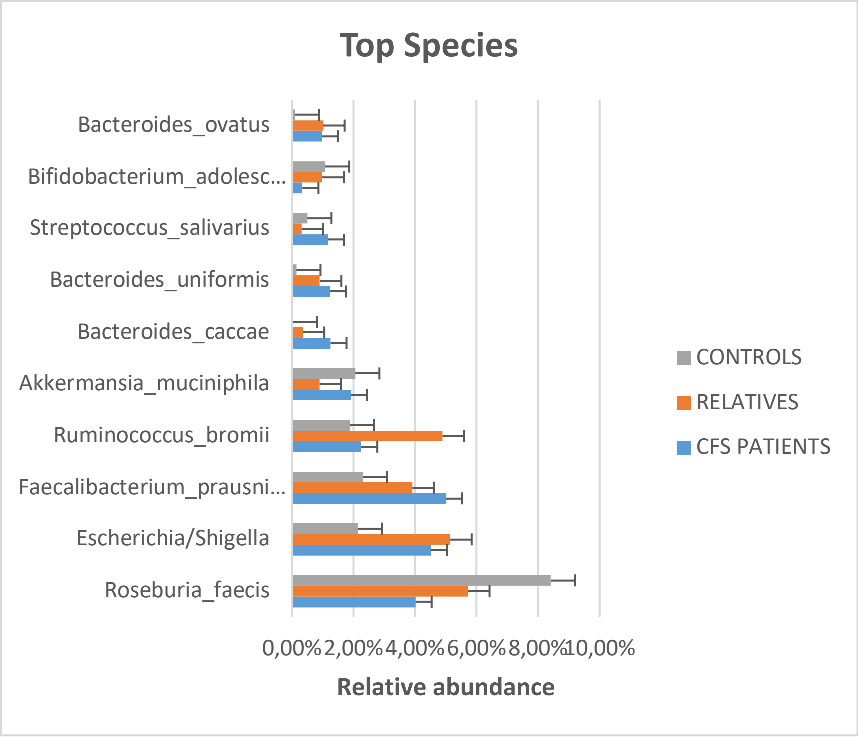

Figure 5. Compositions of the intestinal microbiota from CFS patients, relatives and controls at species level. Only taxa ≥1% in at least one sample are shown.

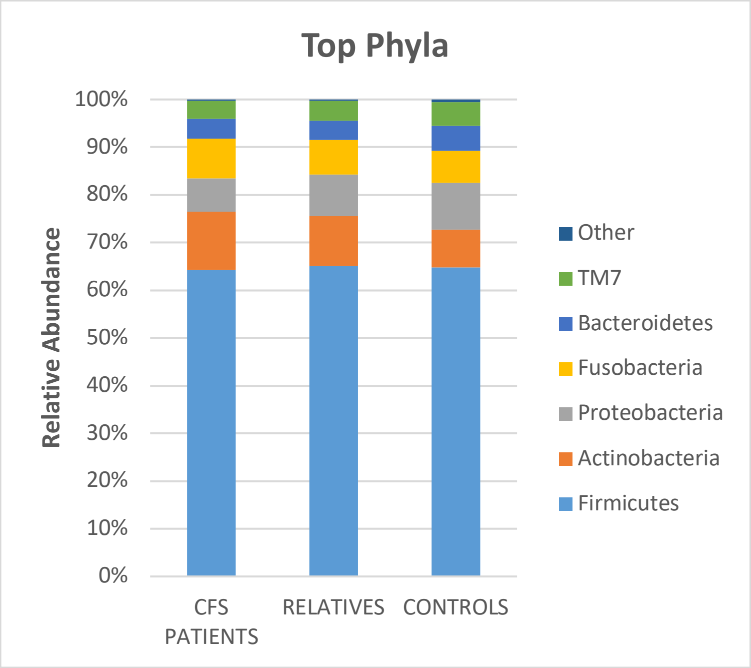

Figure 6. Compositions of the oral microbiota from CFS patients, relatives and controls at phylum level. Only taxa ≥1% in at least one sample are shown, while taxa with lower participations were added to the “other” sequence group.

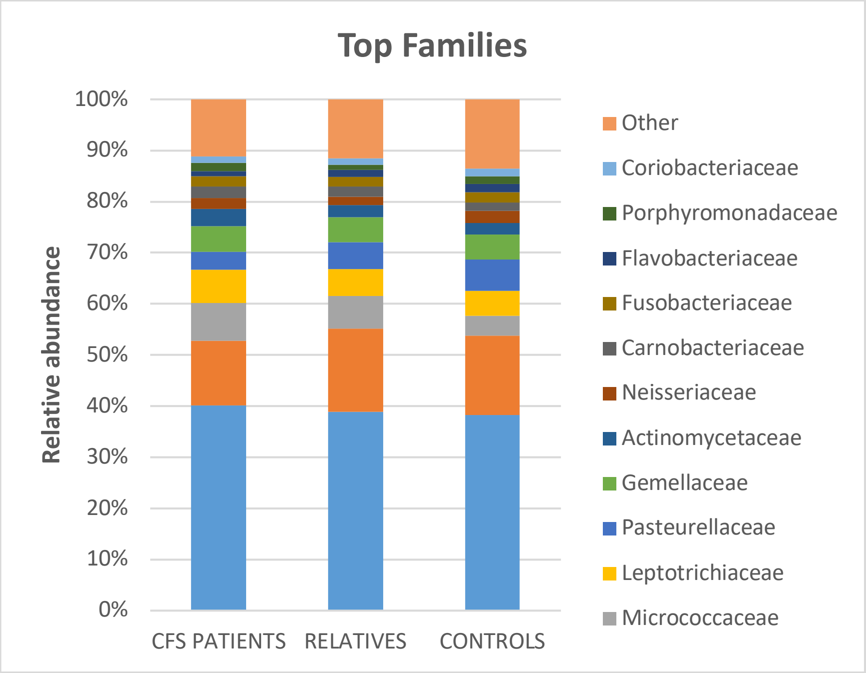

Figure 7. Compositions of the oral microbiota from CFS patients, relatives and controls at family level. Only taxa ≥1% in at least one sample are shown, while taxa with lower participations were added to the “other” sequence group.

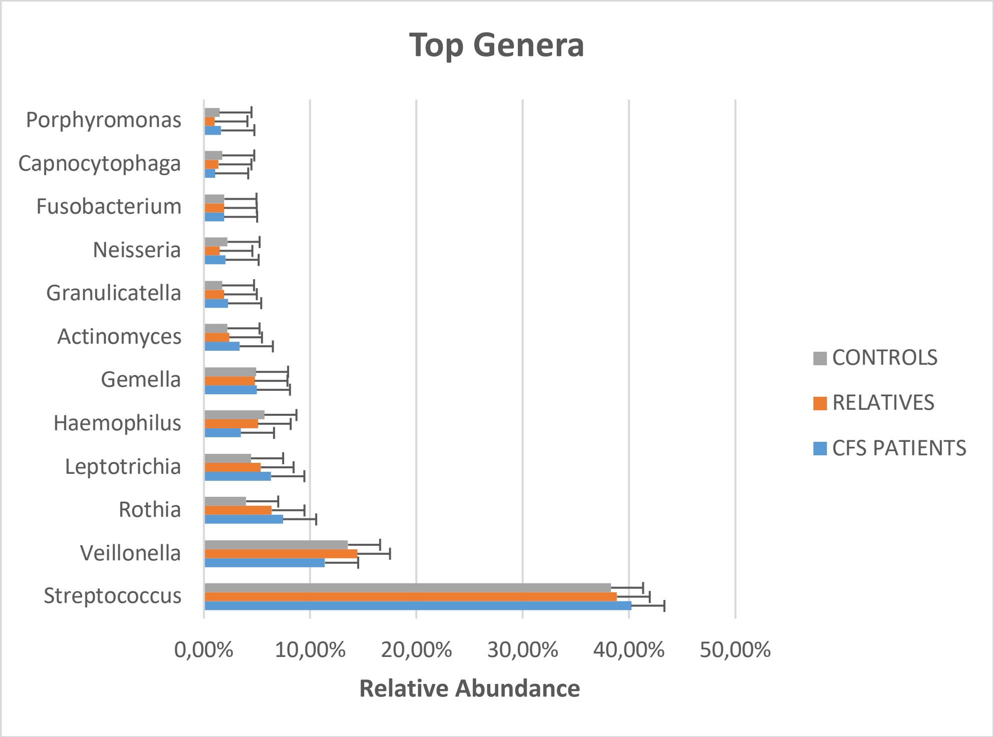

Figure 8. Compositions of the oral microbiota from CFS patients, relatives and controls at genus level. Only taxa ≥1% in at least one sample are shown.

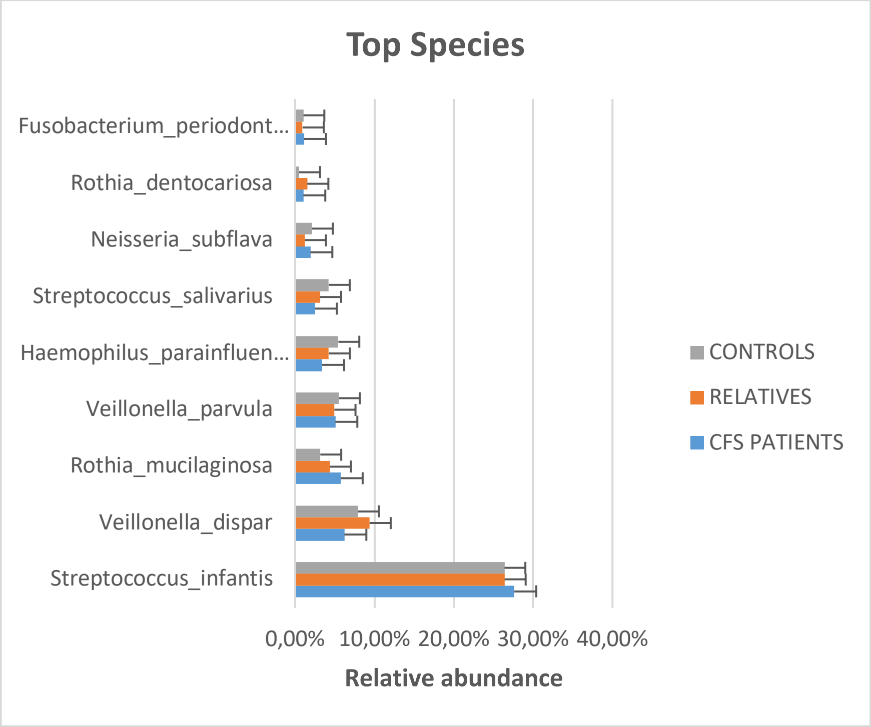

Figure 9. Compositions of the oral microbiota from CFS patients, relatives and controls at species level. Only taxa ≥1% in at least one sample are shown.

Compounds	Class	Composite mass spectrum
**Trigonelline**	Alkaloids	(137.04515, 31546.615)(138.05145, 2701.955)(121.03868, 711.66254)(140.37044, 37125.758)(140.5623, 5581.7725)(165.05385, 1356.0842)
**3-Hydroxyquinine**	Alkaloids	(324.505, 37052.617)(325.50632, 3264.578)(327.18936, 4144.065)
**L-Leucine**	Amino acids, peptides, and analogues	(113.08176, 47147.883)(132.10138, 58039.8)(133.1045, 2105.74)(114.090546, 443.96002)(154.08571, 1013.75415)(136.07436, 3468.0396)(137.0682, 281.18)
**L-Alloisoleucine**	Amino acids, peptides, and analogues	(113.08176, 47147.883)(132.10138, 58373.734)(133.1045, 2105.74)(114.090546, 548.735)(154.08571, 1013.75415)(136.07436, 3502.2732)(137.0682, 281.18)
**L-Isoleucine**	Amino acids, peptides, and analogues	(113.08176, 47147.883)(132.10138, 59172.105)(133.1045, 2144.241)(114.090546, 548.735)(154.08571, 1013.75415)(136.07436, 3606.8892)(137.0682, 281.18)
**L-Norleucine**	Amino acids, peptides, and analogues	(113.5858, 23657.564)(132.10138, 56935.395)(133.1045, 2105.74)(114.090546, 443.96002)(154.08571, 963.7342)(136.07436, 3300.4104)(137.0682, 281.18)
**L-Proline**	Amino acids, peptides, and analogues	(116.069824, 22234.762)(138.0527, 39626.117)(139.0563, 8655.09)
**L-Valine**	Amino acids, peptides, and analogues	(118.08567, 17674.57)(100.07483, 667.6333)(140.06975, 5370.075)(141.0659, 828.22)
**Betaine**	Amino acids, peptides, and analogues	(118.085686, 20091.367)(100.0746, 216.94)(140.06975, 5525.3413)(141.0659, 828.22)
**Ornithine**	Amino acids, peptides, and analogues	(118.0858, 44028.934)(155.08057, 9234.296)(137.06836, 2908.255)
**Aminomalonic acid**	Amino acids, peptides, and analogues	(120.023056, 16915.32)(102.01284, 48839.234)(124.1144, 14872.795)(124.90162, 4788.7036)(125.03705, 6329.73)(102.021034, 5403.425)(147.0225, 3120.546)

**2,4-Diaminobutyric acid**	Amino acids, peptides, and analogues	(120.08015, 152394.92)(121.08324, 6704.0034)(120.08076, 213502.14)(121.08379, 11449.81)(141.06528, 31479.172)(142.0683, 2962.3599)
**L-Threonine**	Amino acids, peptides, and analogues	(121.06413, 2482.47)(103.05367, 11083.381)(123.4066, 8453.51)(146.05907, 1342.4762)(124.03889, 16727.947)
**L-Homoserine**	Amino acids, peptides, and analogues	(121.06413, 2593.2244)(103.05367, 10686.738)(123.4066, 8318.759)(146.05907, 1314.3499)(124.03889, 16743.61)
**L-Glutamic acid**	Amino acids, peptides, and analogues	(130.0486, 2211.33)(174.0538, 5280.61)(175.0566, 111.2)(130.0492, 807.385)(148.0591, 6024.13)(152.19363, 19257.977)(153.0325, 15991.823)
**Pyroglutamic acid**	Amino acids, peptides, and analogues	(130.04918, 3862.1777)(152.33511, 19701.377)(153.03252, 16261.735)(138.02702, 8441.784)
**L-Lysine**	Amino acids, peptides, and analogues	(147.1121, 7929.69)(132.10178, 75690.6)(133.10466, 1793.14)(147.11487, 9162.998)(154.09058, 1269.4451)
**2-Furoylglycine**	Amino acids, peptides, and analogues	(152.03625, 23022.973)(153.0328, 18721.076)(175.01762, 2755.16)
**L-Phenylalanine**	Amino acids, peptides, and analogues	(152.0798, 6620.7)(188.06975, 36042.934)(189.07266, 1319.6941)(166.08565, 93143.05)(167.08842, 3757.1594)(168.101, 429.59)(170.05725, 6850.6626)
**Hexanoylglycine**	Amino acids, peptides, and analogues	(156.76404, 598.70667)(175.11757, 678.10394)(196.09572, 8751.42)(178.08482, 3351.8853)
**Isovalerylglycine**	Amino acids, peptides, and analogues	(159.59314, 2143.5476)(143.08011, 503.41998)(162.10123, 16433.857)(182.08032, 7737.927)(164.57031, 12848.18)
**Citrulline**	Amino acids, peptides, and analogues	(179.53223, 6283.2505)(180.10126, 51499.7)(158.09563, 19417.03)(159.0982, 667.265)(198.09045, 84098.31)(199.09329, 5028.355)
**L-Tyrosine**	Amino acids, peptides, and analogues	(181.40788, 7237.8047)(186.0856, 620.75)(167.07306, 884.34)(182.08029, 4268.3857)(164.06985, 15656.716)
**DL-2-Aminooctanoic acid**	Amino acids, peptides, and analogues	(182.11607, 1950.35)(183.1115, 524.37)(144.2728, 1104.5643)(160.13274, 309046.4)(161.13605, 25783.879)(164.9962, 3847.273)
**N-Acetyl-L-phenylalanine**	Amino acids, peptides, and analogues	(189.07324, 11934.205)(215.0756, 862.775)
**Phenylpropionylglycine**	Amino acids, peptides, and analogues	(189.07324, 11934.205)(215.0756, 862.775)
**Cysteine-S-sulfate**	Amino acids, peptides, and analogues	(200.97273, 8157.806)(201.9738, 547.2217)(202.97108, 616.38)(182.96175, 787.00415)(207.9852, 20468.727)
**L-Tryptophan**	Amino acids, peptides, and analogues	(205.09753, 28117.791)(206.1, 777.19)(205.0985, 21840.21)(192.10086, 526.77)(227.0806, 39637.81)(228.08331, 1105.04)
**L-Acetylcarnitine**	Amino acids, peptides, and analogues	(208.1318, 3353.5266)(227.60953, 10907.76)(230.31137, 6841.37)(208.09592, 17860.455)
**Argininosuccinic acid**	Amino acids, peptides, and analogues	(313.10556, 5277.32)(295.09506, 11714.685)
**Urocanic acid**	Azoles	(123.22534, 14868.759)(124.03895, 19148.504)(161.0286, 35324.055)(162.02951, 4638.645)(163.02605, 3483.01)(122.710396, 903.3867)(139.04945, 18773.39)
**Imidazolelactic acid**	Azoles	(139.04897, 4724.6367)(139.04951, 28753.668)(183.85452, 2099.174)(161.0286, 35324.055)(162.02951, 4638.645)(163.02605, 3483.01)
**Imidazolepropionic acid**	Azoles	(126.05385, 857.92505)(141.06535, 39458.785)(142.0683, 2962.3599)(166.05623, 2135.7979)
**Tyramine**	Benzene and substituted derivatives	(160.07504, 4570.7524)(120.08029, 178789.67)(121.08341, 7242.7812)(138.09035, 5513.475)
**Salicylic acid**	Benzoic acids and derivatives	(138.02719, 1735.6417)(120.02307, 32072.316)(125.03762, 997.7654)(147.02245, 5452.0005)
**Homogentisic acid**	Benzoic acids and derivatives	(151.03502, 187046.89)(152.03618, 19538.54)(153.0355, 2272.81)
**gamma-CEHC**	Benzopyrans	(264.1411, 1696.19)(269.1123, 15903.99)
**N1,N12-Diacetylspermine**	Carboximidic acids and derivatives	(287.24335, 13960.275)(294.21667, 2299.615)
**Palmitoylethanolamide**	Carboximidic acids and derivatives	(323.13004, 5380.7495)(282.27905, 138406.67)(283.27637, 36710.36)(284.2691, 8800.18)(300.28934, 99417.695)(301.29227, 13512.826)(302.30115, 862.9267)

**Methylmalonic acid**	Carboxylic acids and derivatives	(123.040085, 103183.2)(102.020966, 10012.258)(101.02491, 8787.14)(102.0211, 8010.6187)(128.01898, 192560.98)
**Succinic acid**	Carboxylic acids and derivatives	(123.040085, 103183.2)(102.020966, 10012.258)(101.02491, 8787.14)(102.0211, 8010.6187)(128.01898, 192560.98)
**N-Acetylcadaverine**	Carboxylic acids and derivatives	(145.13347, 94327.805)(146.13533, 1735.2965)
**Aminocaproic acid**	Fatty Acyls	(113.5858, 23657.564)(132.10136, 56802.633)(133.1045, 2105.74)(114.090546, 443.96002)(154.08571, 870.66174)(136.07436, 3509.6912)(137.0682, 281.18)
**3-Hydroxyisovaleric acid**	Fatty Acyls	(118.064156, 1758.7616)(123.040245, 46151.133)(124.040695, 5342.8433)(125.03741, 4862.107)
**2-Hydroxy-3-methylbutyric acid**	Fatty Acyls	(118.06417, 1718.7689)(123.04022, 53380.402)(124.040855, 5927.566)(125.03742, 5043.9736)
**2-Ethylhydracrylic acid**	Fatty Acyls	(118.06417, 1718.7689)(123.04022, 53380.402)(124.040855, 5927.566)(125.03742, 5043.9736)
**2-Hydroxyvaleric acid**	Fatty Acyls	(118.06417, 1817.6693)(123.04023, 45623.758)(124.04074, 5599.828)(125.03738, 4937.3647)
**3-Hydroxyvaleric acid**	Fatty Acyls	(118.0642, 1935.1437)(123.040245, 51482.62)(124.04084, 5975.225)(125.03741, 5096.589)
**3-Methylthiopropionic acid**	Fatty Acyls	(120.02306, 32396.521)(102.0127, 91294.78)(130.00746, 116739.98)
**2-Oxo-4-methylthiobutanoic**	Fatty Acyls	(130.00768, 95860.86)(158.00273, 314609.53)
**4-Trimethylammoniobutanoic acid**	Fatty Acyls	(130.1085, 3212947.5)(131.1109, 138886.53)(168.10095, 2257.6052)(128.10655, 3287.2266)(146.11702, 187984.81)(147.11806, 9239.8)(148.1221, 508.01)(150.66267, 5243.472)
**trans-trans-Muconic acid**	Fatty Acyls	(145.03398, 134.8568)(127.0233, 457.24237)
**Methylglutaric acid**	Fatty Acyls	(146.39275, 68490.38)(147.06233, 4983.4062)(148.05919, 750.4634)(128.59404, 4566.0957)(151.03458, 62159.715)(152.03629, 15820.572)(153.03287, 16058.488)
**2-Methylglutaric acid**	Fatty Acyls	(146.39275, 68490.38)(147.06233, 4983.4062)(148.05919, 750.4634)(128.59404, 4566.0957)(151.03458, 62159.715)(152.03629, 15820.572)(153.03287, 16058.488)
**Adipic acid**	Fatty Acyls	(146.39275, 68490.38)(147.06233, 4983.4062)(148.05919, 750.4634)(128.59404, 4566.0957)(151.03458, 62159.715)(152.03629, 15820.572)(153.03287, 16058.488)
**Mevalonic acid**	Fatty Acyls	(148.07527, 115562.48)(149.07855, 10833.09)(130.0643, 1199.51)(153.0898, 631.53503)
**(R)-2-Hydroxycaprylic acid**	Fatty Acyls	(165.09105, 78874.984)(166.09395, 1975.3799)
**Suberic acid**	Fatty Acyls	(174.0904, 22356.514)(175.0929, 365.325)(181.2729, 1086.545)
**Azelaic acid**	Fatty Acyls	(188.10614, 14286.302)(213.1006, 3495.71)(195.08707, 16990.262)(196.09, 1205.775)
**Dodecanedioic acid**	Fatty Acyls	(230.15706, 2319.8455)(217.1538, 1183.0333)(216.90527, 4149.4814)(217.15436, 1188.53)(254.137, 2683.5)(238.5904, 1331.6927)
**1,11-Undecanedicarboxylic acid**	Fatty Acyls	(231.1683, 2231.575)(231.16965, 3864.71)(251.64752, 1917.415)
**Myristoleic acid**	Fatty Acyls	(231.16983, 48202.29)(232.17221, 1399.654)
**Traumatic acid**	Fatty Acyls	(232.91705, 691.22)(233.1481, 723.83)(214.30144, 757.77167)(215.13855, 509.66498)(214.33633, 8980.89)(215.1379, 2627.2202)(253.38211, 3457.88)(236.98975, 2017.4921)
**Heptadecanoic acid**	Fatty Acyls	(253.25241, 105653.93)(254.25519, 9697.025)(253.253, 131569.81)(254.25525, 14728.045)(293.24744, 49315.09)(294.25018, 3827.79)
**Oleamide**	Fatty Acyls	(264.26746, 40842.92)(265.2695, 3649.51)(282.2795, 349594.66)(283.27176, 133120.67)(284.2827, 2315.98)(282.27625, 14474.15)
**alpha-Linolenic acid**	Fatty Acyls	(279.10513, 139776.62)(280.23465, 18159.09)(281.23825, 1682.845)(279.23154, 18118.158)(304.22986, 1265.685)
**Palmitic acid**	Fatty Acyls	(279.23166, 156262.17)(280.2346, 17831.746)(281.23825, 1682.845)
**Linoleic acid**	Fatty Acyls	(281.09174, 201605.69)(282.25046, 31345.305)(263.23618, 28894.057)(264.23877, 2613.2415)(281.24713, 32319.408)(282.2505, 3778.3691)(263.2367, 51336.004)(264.2396, 4271.279)
**Elaidic acid**	Fatty Acyls	(282.813, 97504.625)(284.26538, 17102.938)(264.97064, 84887.65)(266.25543, 18057.025)(267.76056, 799.31995)(283.3763, 201154.16)(284.26743, 51690.312)(285.26907, 2458.0234)(265.25235, 107820.33)(266.25555, 19372.719)(309.25687, 2592.9314)
**Oleic acid**	Fatty Acyls	(282.813, 97504.625)(284.26538, 17102.938)(264.97064, 84887.65)(266.25543, 18057.025)(267.76056, 799.31995)(283.3763, 201154.16)(284.26743, 51690.312)(285.26907, 2458.0234)(265.25235, 107820.33)(266.25555, 19372.719)(309.25687, 2592.9314)
**Stearic acid**	Fatty Acyls	(284.26697, 38604.363)(285.26907, 2903.6233)(266.25558, 19979.738)(267.25897, 742.595)(288.00104, 3678.9087)(289.292, 318.675)(310.2725, 281.27502)
**Octadecanedioic acid**	Fatty Acyls	(297.1065, 6597.3325)(297.242, 21523.584)(298.24597, 8950.39)(319.2235, 11890.111)(320.228, 1198.6599)
**Eicosapentaenoic acid**	Fatty Acyls	(307.20102, 101534.02)(308.20367, 9882.461)
**Prostaglandin F2a**	Fatty Acyls	(336.22223, 2066.955)(337.2369, 81274.5)(338.23932, 7045.685)
**Adrenic acid**	Fatty Acyls	(355.26273, 204910.67)(356.2657, 39930.13)(337.25003, 17160.94)(338.25455, 3296.2905)

**Prostaglandin F1a**	Fatty Acyls	(356.26553, 21700.38)(338.2544, 2685.7148)(339.2578, 28589.172)(340.26508, 2856.145)(380.24152, 344.75)
**L-Palmitoylcarnitine**	Fatty Acyls	(383.325, 2381.915)(385.3412, 10384.625)(399.54468, 127111.33)(400.3515, 25142.016)(401.3562, 9014.46)(400.68774, 7839.5024)(401.3569, 1917.35)(407.32358, 2025.135)
**Sumiki's acid**	Furans	(145.03398, 134.8568)(127.0233, 457.24237)
**Glycerylphosphorylethanolamine**	Glycerophospholipids	(216.0534, 5789.295)(198.05188, 2741.7832)(198.05249, 85910.86)(199.05515, 1673.3301)(200.05075, 1604.285)
**PC(18:1(9Z)/18:1(9Z))**	Glycerophospholipids	(785.5917, 327288.2)(786.5948, 164409.4)(787.5978, 38835.047)(788.6, 4218.135)(767.5801, 6905.622)(768.5834, 2995.2854)(808.5761, 37836.633)(809.57434, 6793.1494)
**3-Hydroxyoctanoic acid**	Hydroxy acids and derivatives	(165.09105, 78874.984)(166.09395, 1975.3799)
**7-Hydroxyoctanoic acid**	Hydroxy acids and derivatives	(165.09105, 78874.984)(166.09395, 1975.3799)
**Hydroxyoctanoic acid**	Hydroxy acids and derivatives	(165.09105, 78874.984)(166.09395, 1975.3799)
**Hypoxanthine**	Imidazopyrimidines	(137.04515, 29810.482)(138.05145, 2574.125)(122.754265, 9193.555)(123.8042, 13487.565)(124.0391, 31486.172)(161.02861, 36982.65)(162.02945, 7781.555)
**Xanthine**	Imidazopyrimidines	(152.03616, 24351.89)(153.03271, 20462.93)(138.02689, 9560.74)(153.0387, 8697.13)(154.049, 1478.7)(175.01791, 3218.5146)
**8-Hydroxyguanine**	Imidazopyrimidines	(168.05013, 8308.795)(151.03522, 271077.8)(152.03639, 30015.615)(168.0509, 6236.89)
**1,3,7-Trimethyluric acid**	Imidazopyrimidines	(213.33423, 1410.7175)(192.0648, 24748.498)
**Indoleacrylic acid**	Indoles and derivatives	(170.0588, 937.7225)(214.06424, 3572.1436)(188.06989, 60806.816)(189.07262, 3362.656)
**Indolelactic acid**	Indoles and derivatives	(188.06989, 63381.293)(189.07262, 3351.06)(214.06422, 3250.822)
**5-Hydroxyindoleacetic acid**	Indoles and derivatives	(214.04703, 4426.9316)(174.0538, 2306.4248)(192.0648, 27854.559)
**Pyruvic acid**	Keto acids and derivatives	(112.0065, 89022.98)(115.0109, 5849.04)
**Oxoglutaric acid**	Keto acids and derivatives	(148.35997, 77543.49)(151.03519, 248238.1)(129.02032, 20066.727)(130.0163, 15295.64)
**L-Pipecolic acid**	L-Pipecolic acid	(130.08557, 17026.27)(113.08145, 4084.085)(152.44482, 455.11746)(134.797, 62657.027)(135.24463, 3423.8188)
**Naproxen**	Naphthalenes	(214.08525, 5624.4697)(217.09616, 1442.015)
**Choline**	Organonitrogen compounds	(104.1067, 78783.47)(105.1093, 297.92)
**1-Phenylethylamine**	Organonitrogen compounds	(144.0796, 4112.2573)(122.095604, 42402.445)(123.09867, 1966.1421)
**L-Carnitine**	Organonitrogen compounds	(162.11157, 1625.3153)(144.1013, 1455.535)(166.122, 2255.0151)(144.43523, 165925.3)(145.10455, 25304.225)(186.77122, 1241.5435)(166.08562, 66723.42)(167.08855, 2655.1997)(168.101, 271.32)
**Sphingosine**	Organonitrogen compounds	(323.13004, 5380.7495)(282.27905, 137055.52)(283.27637, 36710.36)(284.2691, 8800.18)(300.28934, 98528.19)(301.29227, 13430.815)(302.30115, 862.9267)
**Sphinganine**	Organonitrogen compounds	(324.28885, 14428.993)(325.2919, 2202.6724)(284.29382, 55714.46)(285.2967, 4784.8423)(302.25125, 76237.48)(303.67062, 17523.031)(304.31125, 1842.8201)(302.305, 193784.75)(303.3079, 36507.227)(304.3101, 1760.61)(307.78333, 1553.325)
**Glyceric acid**	Organooxygen compounds	(130.0163, 13551.984)(112.00613, 61977.445)(115.0109, 5849.04)
**Ribitol**	Organooxygen compounds	(134.05945, 110360.43)(135.06233, 3615.3772)(175.05695, 13723.205)
**L-Arabitol**	Organooxygen compounds	(134.05945, 110360.43)(135.06233, 3615.3772)(175.05695, 13723.205)
**D-Arabitol**	Organooxygen compounds	(134.05946, 110895.34)(135.06235, 3620.3108)(175.0569, 12996.82)
**4-Hydroxycyclohexylcarboxylic acid**	Organooxygen compounds	(144.0797, 6953.6743)(130.08522, 8480.647)(149.0589, 9117.347)
**trans-4-Hydroxycyclohexylacetic acid**	Organooxygen compounds	(158.0957, 22461.35)(159.0982, 678.02)(144.10095, 14968.418)(166.08537, 2527.9624)
**cis-4-Hydroxycyclohexylacetic acid**	Organooxygen compounds	(158.0957, 22461.35)(159.0982, 678.02)(144.10095, 14968.418)(166.08537, 2527.9624)
**Threonic acid**	Organooxygen compounds	(161.02861, 36982.65)(162.02945, 7781.555)(123.04057, 14925.162)(137.04514, 28540.014)(138.05145, 2574.125)
**Erythronic acid**	Organooxygen compounds	(161.02861, 36982.65)(162.02945, 7781.555)(123.04067, 16288.55)(137.04515, 29810.482)(138.05145, 2574.125)
**L-Fucose**	Organooxygen compounds	(164.0695, 5227.252)(146.05942, 61165.824)(147.0625, 5008.407)(148.0593, 480.20667)(192.06494, 18675.482)
**Rhamnose**	Organooxygen compounds	(164.0695, 5451.253)(146.05942, 61297.207)(147.0625, 5008.407)(148.0593, 480.20667)(192.06494, 18675.482)
**Mannitol**	Organooxygen	(182.08058, 4133.0957)(164.57045, 12178.269)

	compounds	
**Sorbitol**	Organooxygen compounds	(182.08058, 4277.184)(164.57045, 12553.081)
**Galactitol**	Organooxygen compounds	(182.08058, 4277.184)(164.57045, 12553.081)
**Pantothenic acid**	Organooxygen compounds	(220.11833, 25050.184)(223.69765, 2329.3494)(224.12726, 5824.945)(220.1177, 10207.93)(224.0902, 1196.47)
**Glucose 6-phosphate**	Organooxygen compounds	(265.34946, 23632.537)(266.34894, 6410.4077)(267.01312, 1492.33)
**myo-Inositol 1-phosphate**	Organooxygen compounds	(265.34946, 23632.537)(266.34894, 6410.4077)(267.01312, 1492.33)
**Mannose 6-phosphate**	Organooxygen compounds	(265.34946, 23632.537)(266.34894, 6410.4077)(267.01312, 1492.33)
**3-Dehydrosphinganine**	Organooxygen compounds	(323.13004, 5380.7495)(282.27905, 137055.52)(283.27637, 36710.36)(284.2691, 8800.18)(300.28934, 98528.19)(301.29227, 13430.815)(302.30115, 862.9267)
**Carnosine**	Peptidomimetics	(227.42168, 10915.369)(230.31137, 6841.37)(208.09592, 17860.455)(230.11627, 11993.194)(231.11855, 426.19)(210.11124, 8078.2783)(236.10156, 1621.706)
**Benzoic acid**	Phenolic acids	(123.04007, 83904.62)(124.041, 6767.267)(128.01894, 154775.14)(129.02031, 17085.447)
**Acetaminophen**	Phenolic acids	(134.05945, 91109.62)(135.06242, 4153.413)(174.05373, 30908.41)(175.05685, 4673.1104)
**2-Aminobenzoic acid**	Phenolic acids	(137.04515, 31546.615)(138.05145, 2701.955)(121.03868, 711.66254)(140.37044, 37125.758)(140.5623, 5581.7725)(165.05385, 1356.0842)
**p-Aminobenzoic acid**	Phenolic acids	(137.04515, 31546.615)(138.05145, 2701.955)(121.03868, 711.66254)(140.37044, 37125.758)(140.5623, 5581.7725)(165.05385, 1356.0842)
**3-Hydroxybenzoic acid**	Phenolic acids	(138.02719, 1616.2399)(120.023056, 31552.473)(125.0376, 1035.5084)(147.02245, 5319.3574)
**4-Hydroxybenzoic acid**	Phenolic acids	(138.02719, 1665.5927)(120.02307, 31631.924)(125.03759, 1020.20306)(147.02245, 5366.685)
**3-Hydroxymandelic acid**	Phenolic acids	(151.03502, 187046.89)(152.03618, 19538.54)(153.0355, 2272.81)
**p-Hydroxymandelic acid**	Phenolic acids	(151.03502, 187046.89)(152.03618, 19538.54)(153.0355, 2272.81)
**3,4-Dihydroxybenzeneacetic acid**	Phenolic acids	(151.03502, 187046.89)(152.03618, 19538.54)(153.0355, 2272.81)
**Vanillic acid**	Phenolic acids	(151.03502, 187170.36)(152.03618, 19546.592)(153.0355, 2272.81)
**Cinnamic acid**	Phenolic acids	(152.0682, 461.63)(136.0612, 6453.17)(174.0538, 5077.51)(175.0566, 106.92)
**Normetanephrine**	Phenols	(166.08502, 44556.81)(167.08772, 1988.38)(188.10486, 151.25)(185.77, 941.6767)(188.06996, 47874.11)(189.07278, 2368.1445)
**Vitamin A**	Prenol lipids	(287.24335, 13960.275)(294.21667, 2299.615)
**Phytol**	Prenol lipids	(301.2923, 57092.168)(302.29654, 1510.614)
**gamma-Tocopherol**	Prenol lipids	(400.356, 100740.18)(401.35898, 20153.006)(402.3625, 1839.2375)
**alpha-Tocopherol**	Prenol lipids	(430.376, 5997.5786)(435.35947, 4357.29)
**Oleanolic acid**	Prenol lipids	(457.3641, 41206.74)(458.36688, 6897.95)(457.36652, 112405.67)(458.36957, 26033.41)(459.3735, 3427.565)(480.3518, 19713.521)(481.35428, 1900.9911)(463.23648, 5093.8)(465.3578, 215.825)
**Ursolic acid**	Prenol lipids	(457.3641, 41206.74)(458.36688, 6897.95)(457.36652, 112405.67)(458.36957, 26033.41)(459.3735, 3427.565)(480.3518, 19713.521)(481.35428, 1900.9911)(463.23648, 5093.8)(465.3578, 215.825)
**Picolinic acid**	Pyridines and derivatives	(124.039215, 16054.716)(125.03728, 6166.8677)(150.76704, 30170.445)(151.03455, 219558.58)(130.01614, 12028.521)
**Nicotinic acid**	Pyridines and derivatives	(124.039215, 16323.262)(125.03728, 6166.8677)(150.76704, 30031.125)(151.03455, 219558.58)(130.01614, 12028.521)
**Niacinamide**	Pyridines and derivatives	(145.03395, 3033.815)(127.02333, 12256.792)
**Pyridoxine**	Pyridines and derivatives	(174.0897, 12075.171)(174.09047, 30689.604)(175.0929, 425.63498)(192.06477, 22210.018)(174.05382, 63675.062)
**Tetrahydrodeoxycorticosterone**	Steroids and steroid derivatives	(335.25534, 87493.836)(336.25815, 8897.667)
**3b-Hydroxy-5-cholenoic acid**	Steroids and steroid derivatives	(374.27567, 1822.6748)(356.40997, 26236.775)(358.27997, 28164.822)(359.2806, 269.07)(375.28787, 4386.765)(357.279, 748884.56)(358.2821, 176478.27)(359.2844, 12670.022)
**Lithocholic acid**	Steroids and steroid derivatives	(376.29092, 2877.26)(358.57632, 23703.234)(360.29666, 10166.264)(379.6426, 5048.665)(381.31818, 1803.01)(359.29425, 205453.81)(360.29733, 40086.2)(361.29993, 3900.265)
**Isolithocholic acid**	Steroids and steroid derivatives	(376.29092, 2877.26)(358.57632, 23703.234)(360.29666, 10166.264)(379.6426, 5048.665)(381.31818, 1803.01)(359.29425, 205453.81)(360.29733, 40086.2)(361.29993, 3900.265)
**24-Hydroxycholesterol**	Steroids and steroid derivatives	(385.346, 53229.47)(386.34897, 8327.909)
**7alpha-Hydroxycholesterol**	Steroids and steroid derivatives	(385.346, 53229.47)(386.34897, 8327.909)

**Cholestenone**	Steroids and steroid derivatives	(385.34604, 57044.598)(386.34897, 9035.905)(369.3511, 1605.6075)
**3,7-Dihydroxy-12-oxocholanoic acid**	Steroids and steroid derivatives	(389.26715, 19466.691)(390.27023, 3814.4766)(411.45078, 14079.596)(413.26016, 5402.41)
**7-Ketodeoxycholic acid**	Steroids and steroid derivatives	(389.26718, 18307.375)(390.27023, 3814.4766)(411.4326, 12889.281)(413.26016, 5402.41)
**3-Oxocholic acid**	Steroids and steroid derivatives	(389.26718, 18307.375)(390.27023, 3814.4766)(411.4326, 12889.281)(413.26016, 5402.41)
**Cholic acid**	Steroids and steroid derivatives	(390.27023, 2807.452)(414.29764, 4084.67)(391.28397, 259978.69)(392.28677, 51298.94)(393.2891, 3357.6848)(413.2654, 89416.59)(414.26865, 13560.644)
**Hyocholic acid**	Steroids and steroid derivatives	(390.27023, 2807.452)(414.29764, 4084.67)(391.28397, 259978.69)(392.28677, 51298.94)(393.2891, 3357.6848)(413.2654, 89416.59)(414.26865, 13560.644)
**3a,6b,7b-Trihydroxy-5b-cholanoic acid**	Steroids and steroid derivatives	(390.27023, 2807.452)(414.29764, 4084.67)(391.28397, 259978.69)(392.28677, 51298.94)(393.2891, 3357.6848)(413.2654, 89416.59)(414.26865, 13560.644)
**Nutriacholic acid**	Steroids and steroid derivatives	(390.27023, 2880.925)(372.8225, 32776.78)(374.27567, 9249.087)(375.28516, 955.49)(391.28397, 262341.1)(392.28677, 51822.58)(393.2891, 3402.9197)(373.27295, 88699.86)(374.2758, 14285.864)(375.28192, 1763.77)(413.2654, 90171.82)(414.26865, 13688.092)(415.27362, 305.87)
**Isoursodeoxycholic acid**	Steroids and steroid derivatives	(397.3085, 733.48)(374.53973, 2647.5115)(375.28778, 6517.1743)(415.2812, 68583.09)(416.28403, 10314.268)(417.2868, 1475.22)
**Isodeoxycholic acid**	Steroids and steroid derivatives	(397.3085, 733.48)(374.53973, 2647.5115)(375.28778, 6517.1743)(415.2812, 68583.09)(416.28403, 10314.268)(417.2868, 1475.22)
**Deoxycholic acid**	Steroids and steroid derivatives	(397.3085, 733.48)(374.53973, 2647.5115)(375.28778, 6517.1743)(415.2812, 68583.09)(416.28403, 10314.268)(417.2868, 1475.22)
**Chenodeoxycholic acid**	Steroids and steroid derivatives	(397.3085, 733.48)(374.53973, 2647.5115)(375.28778, 6517.1743)(415.2812, 68583.09)(416.28403, 10314.268)(417.2868, 1475.22)
**Ursodeoxycholic acid**	Steroids and steroid derivatives	(397.3085, 733.48)(374.53973, 2647.5115)(375.28778, 6517.1743)(415.2812, 68583.09)(416.28403, 10314.268)(417.2868, 1475.22)
**Ergosterol**	Steroids and steroid derivatives	(398.34103, 331890.62)(399.52805, 92027.22)(400.47998, 75606.04)(401.35944, 32194.21)(402.36334, 8897.029)
**Cholesterol**	Steroids and steroid derivatives	(409.34152, 4946.0664)(369.3468, 6837.673)
**Epi-coprostanol**	Steroids and steroid derivatives	(411.36133, 44493.926)(412.36426, 7780.6743)(393.35016, 8398.756)(394.35352, 760.5725)
**5beta-Coprostanol**	Steroids and steroid derivatives	(411.36133, 44493.926)(412.36426, 7780.6743)(393.35016, 8398.756)(394.35352, 760.5725)
**5alpha-Cholestanol**	Steroids and steroid derivatives	(411.36133, 44493.926)(412.36426, 7780.6743)(393.35016, 8398.756)(394.35352, 760.5725)
**Stearoylcarnitine**	Steroids and steroid derivatives	(427.3558, 40287.758)(428.3588, 7686.5786)(429.37305, 32258.469)(430.37604, 6591.868)(456.3663, 4092.6936)(435.35934, 636.0814)
**3alpha,7alpha-Dihydroxycoprostanic acid**	Steroids and steroid derivatives	(435.35864, 7442.228)(457.34058, 4495.4062)
**5b-Cholestane-3a,7a,12a,23S,25-pentol**	Steroids and steroid derivatives	(435.35864, 7442.228)(457.34058, 4495.4062)
**Glycoursodeoxycholic acid**	Steroids and steroid derivatives	(452.3249, 25151.709)(451.323, 9419.85)(455.3319, 2744.86)(432.3096, 75460.14)(433.31238, 12672.087)(472.55368, 19695.084)(473.30518, 4574.4673)(454.291, 33883.45)(455.29535, 4343.475)
**Deoxycholic acid glycine conjugate**	Steroids and steroid derivatives	(452.3249, 25151.709)(451.323, 9419.85)(455.3319, 2744.86)(432.3096, 75460.14)(433.31238, 12672.087)(472.55368, 19695.084)(473.30518, 4574.4673)(454.291, 33883.45)(455.29535, 4343.475)
**Chenodeoxycholic acid glycine conjugate**	Steroids and steroid derivatives	(452.82584, 15775.725)(451.32233, 7936.83)(432.30914, 75111.39)(433.3119, 12625.908)(472.70282, 20149.76)(473.30402, 5374.59)(454.2909, 36493.92)(455.29538, 4539.31)
**Glycocholic acid**	Steroids and steroid derivatives	(466.31482, 68099.21)(467.3174, 11930.519)(448.30396, 19022.916)(449.30652, 5927.295)(488.29404, 18247.299)(489.2913, 3601.3018)(473.29657, 11529.11)
**Ursodeoxycholic acid 3-sulfate**	Steroids and steroid derivatives	(472.2424, 5895.41)(474.26147, 4420.6196)(479.26324, 53143.086)(461.25195, 3773.2)(501.24426, 5756.595)
**Chenodeoxycholic acid 3-sulfate**	Steroids and steroid derivatives	(472.2454, 5269.4136)(474.2606, 14375.021)(477.26233, 28436.855)(479.2635, 64144.242)(461.25214, 4622.435)(498.74243, 5684.7676)(501.24475, 9194.42)
**Lithocholyltaurine**	Steroids and steroid derivatives	(488.30356, 4814.5522)(466.29068, 1972.02)(467.29932, 37179.24)(468.3011, 8120.875)(488.28455, 15882.667)(489.28784, 4129.2974)
**Mesobilirubinogen**	Tetrapyrroles and derivatives	(592.36005, 283480.0)(593.5279, 251217.05)(594.3315, 56119.93)(595.3377, 6272.89)(593.3323, 396976.56)(594.3353, 139423.92)(595.3428, 17090.873)(596.3481, 2189.6252)(616.7576, 25002.516)(616.6261, 10334.538)(617.32104, 1892.1925)

Table 1. Annotated compounds with their composite mass spectrum against Fecal Metabolome database. Classification of compounds is reported in the table.

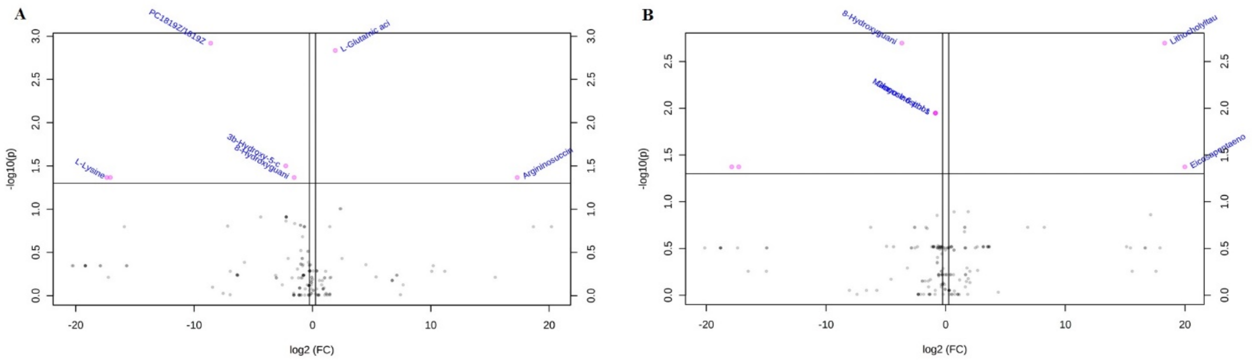

Figure 10. Volcano plot analysis combining one way ANOVA ($p < 0.05$) with the fold changes analysis (cutoff > ± 1.2) of the mass features comparing CFS/ME patients (A) and their relatives (B) to healthy controls.

VIP markers	VIP score	Fold-Change (CFSvsCTR)
**L-Glutamic acid**	3,0293	1,9278
**alpha-Tocopherol**	2,3586	-17,38
**Argininosuccinic acid**	2,3557	17,299
**L-Acetylcarnitine**	2,3336	2,3964
**L-Lysine**	2,327	-17,079
**Carnosine**	2,3208	2,3269
**PC1819Z/1819Z**	2,3071	-8,5994
**8-Hydroxyguanine**	2,3027	-1,5517
**Dodecanedioic acid**	2,0695	-1,5072
**2-Oxo-4-methylthiobutanoic**	2,0308	-2,2381
**Isoursodeoxycholic acid**	1,9774	-2,2186
**Isodeoxycholic acid**	1,9774	-2,2186
**Deoxycholic acid**	1,9774	-2,2186
**Chenodeoxycholic acid**	1,9774	-2,2186
**Ursodeoxycholic acid**	1,9774	-2,2186
**Eicosapentaenoic acid**	1,8257	20,194
**2,4-Diaminobutyric acid**	1,7751	1,4634
**3b-Hydroxy-5-cholenoic acid**	1,7651	-2,2578
**Aminomalonic acid**	1,7549	-4,378
**Lithocholyltaurine**	1,7409	18,667
**Cholesterol**	1,6411	-15,898
**Glycocholic acid**	1,5932	2,4939
**Niacinamide**	1,5825	-0,9848
**Traumatic acid**	1,4878	-2,0565
**gamma-Tocopherol**	1,4838	10,206
**Oleanolic acid**	1,4752	-0,98308
**Ursolic acid**	1,4752	-0,98308
**Phytol**	1,3522	4,5129

**Palmitic acid**	1,2309	-6,9472
**L-Threonine**	1,21	0
**Pyridoxine**	1,195	10,077
**L-Homoserine**	1,1923	0
**5-Hydroxyindoleacetic acid**	1,1869	11,178
**3-Methylthiopropionic acid**	1,1494	-0,83911
**Chenodeoxycholic acid glycine conjugate**	1,1431	1,1138
**2-Furoylglycine**	1,1104	-0,80589
**4-Trimethylammoniobutanoic acid**	1,0997	-7,1671
**Mevalonic acid**	1,0869	-20,299
**Prostaglandin F2a**	1,0857	-20,225
**N-Acetylcadaverine**	1,0609	-19,208
**3-Hydroxyoctanoic acid**	1,0591	-19,194
**7-Hydroxyoctanoic acid**	1,0591	-19,194
**R-2-Hydroxycaprylic acid**	1,0591	-19,194
**Hydroxyoctanoic acid**	1,0591	-19,194
**Lithocholic acid**	1,0314	7,1255
**Isolithocholic acid**	1,0314	7,1255
**trans-4-Hydroxycyclohexylacetic acid**	1,0288	-17,924
**cis-4-Hydroxycyclohexylacetic acid**	1,0288	-17,924
**Ergosterol**	1,0268	-3,1169

Table 2. Annotated metabolites with VIP score > 1 resulting from PLS-DA.

**Markers S-plot**	**p[1]**	**p(corr)[1]**
**L-Glutamic acid**	-10,274	-0,81071
**L-Acetylcarnitine**	-7,9903	-0,60979
**Carnosine**	-7,9262	-0,60625
**Argininosuccinic acid**	-7,8981	-0,65018
**Eicosapentaenoic acid**	-6,0088	-0,48704
**2,4-Diaminobutyric acid**	-5,9563	-0,51364
**Lithocholyltaurine**	-5,733	-0,4862
**Glycocholic acid**	-5,2745	-0,39922
**Oleanolic acid**	-4,9527	-0,37733
**Ursolic acid**	-4,9527	-0,37733
**gamma-Tocopherol**	-4,9313	-0,35399
**Phytol**	-4,6125	-0,37282
**Palmitic acid**	-4,2859	-0,2952
**5-Hydroxyindoleacetic acid**	-4,2125	-0,30145
**Pyridoxine**	-4,1843	-0,29733
**L-Threonine**	-4,0066	-0,36047
**Chenodeoxycholic acid glycine conjugate**	-3,9686	-0,29607
**L-Homoserine**	-3,9447	-0,35469

**Lithocholic acid**	-3,4608	-0,24806
**Isolithocholic acid**	-3,4608	-0,24806
**L-Phenylalanine**	-3,2384	-0,30376
**1-Phenylethylamine**	-3,1047	-0,2962
**Acetaminophen**	-3,0446	-0,22971
**Ribitol**	-2,9079	-0,21928
**L-Arabitol**	-2,9079	-0,21928
**Glycerylphosphorylethanolamine**	-2,7517	-0,23282
**Hypoxanthine**	-2,5126	-0,24305
**Threonic acid**	-2,5126	-0,24305
**Erythronic acid**	-2,5126	-0,24305
**Trigonelline**	-2,5104	-0,242
**2-Aminobenzoic acid**	-2,5104	-0,242
**p-Aminobenzoic acid**	-2,5104	-0,242
**Adrenic acid**	-2,4726	-0,24722
**Xanthine**	-2,372	-0,23759
**2-Hydroxy-3-methylbutyric acid**	-2,1829	-0,24457
**2-Ethylhydracrylic acid**	-2,1829	-0,24457
**Oleamide**	-2,1236	-0,14788
**Cinnamic acid**	-2,1174	-0,24769
**L-Palmitoylcarnitine**	-1,8683	-0,13697
**D-Arabitol**	-1,496	-0,11644
**24-Hydroxycholesterol**	-1,2293	-0,091631
**7alpha-Hydroxycholesterol**	-1,2293	-0,091631
**Hexanoylglycine**	-0,98875	-0,079051
**Cholic acid**	-0,85097	-0,10317
**Hyocholic acid**	-0,85097	-0,10317
**3a,6b,7b-Trihydroxy-5b-cholanoic acid**	-0,8509	-0,10316
**Glycoursodeoxycholic acid**	-0,78661	-0,062701
**Deoxycholic acid glycine conjugate**	-0,78661	-0,062701
**L-Pipecolic acid**	-0,68211	-0,056898
**Urocanic acid**	-0,66607	-0,12898
**Pipecolic acid**	-0,62304	-0,052009
**L-Tyrosine**	-0,61239	-0,048812
**L-Carnitine**	-0,59287	-0,066474
**3-Hydroxyisovaleric acid**	-0,54057	-0,12248
**Pantothenic acid**	-0,39711	-0,031421
**1,3,7-Trimethyluric acid**	-0,33849	-0,028897
**Citrulline**	-0,30993	-0,02545
**Tetrahydrodeoxycorticosterone**	-0,16792	-0,012477
**3,7-Dihydroxy-12-oxocholanoic acid**	-0,049696	-0,004323
**7-Ketodeoxycholic acid**	-0,049696	-0,004323
**3-Oxocholic acid**	-0,049696	-0,004323

**3-Hydroxybenzoic acid**	0,019686	0,0015881
**3-Hydroxyvaleric acid**	0,074484	0,016082
**2-Hydroxyvaleric acid**	0,1003	0,02161
**Cholestenone**	0,129	0,0096724
**Octadecanedioic acid**	0,1709	0,014768
**Ornithine**	0,18899	0,016709
**Betaine**	0,23448	0,020433
**Epi-coprostanol**	0,24095	0,01965
**5beta-Coprostanol**	0,24095	0,01965
**5alpha-Cholestanol**	0,24095	0,01965
**L-Valine**	0,34133	0,029635
**Isovalerylglycine**	0,38319	0,03044
**1,11-Undecanedicarboxylic acid**	0,38452	0,032607
**DL-2-Aminooctanoic acid**	0,43984	0,034333
**Linoleic acid**	0,5088	0,043871
**3alpha,7alpha-Dihydroxycoprostanic acid**	0,54165	0,044294
**5b-Cholestane-3a,7a,12a,23S,25-pentol**	0,54165	0,044294
**Nutriacholic acid**	0,63268	0,074277
**alpha-Linolenic acid**	0,70577	0,049708
**Homogentisic acid**	0,80163	0,29471
**3-Hydroxymandelic acid**	0,80163	0,29471
**p-Hydroxymandelic acid**	0,80163	0,29471
**3,4-Dihydroxybenzeneacetic acid**	0,80163	0,29471
**Oxoglutaric acid**	0,89155	0,29769
**Vanillic acid**	0,96927	0,36029
**Mesobilirubinogen**	0,98528	0,074176
**Imidazolelactic acid**	0,99251	0,073572
**Prostaglandin F1a**	0,99654	0,071328
**Benzoic acid**	1,1035	0,381
**Glyceric acid**	1,2385	0,43873
**Pyroglutamic acid**	1,2815	0,43016
**Stearoylcarnitine**	1,5527	0,12143
**Mannitol**	1,5664	0,13682
**Sorbitol**	1,5664	0,13682
**Galactitol**	1,5664	0,13682
**Methylmalonic acid**	1,5919	0,15266
**Succinic acid**	1,5919	0,15266
**3-Hydroxyquinine**	1,6837	0,15702
**4-Hydroxybenzoic acid**	1,726	0,14716
**trans-trans-Muconic acid**	1,7511	0,14967
**Sumiki's acid**	1,7511	0,14967
**Aminocaproic acid**	1,7774	0,24943
**Elaidic acid**	1,8088	0,20692

**Oleic acid**	1,8088	0,20692
**L-Isoleucine**	1,827	0,2551
**L-Norleucine**	1,8462	0,25821
**L-Alloisoleucine**	1,8823	0,26268
**Salicylic acid**	1,8846	0,16134
**L-Leucine**	1,9021	0,26565
**Picolinic acid**	1,9113	0,40166
**Normetanephrine**	1,9214	0,47375
**Nicotinic acid**	1,9433	0,4092
**Glucose 6-phosphate**	1,9453	0,50021
**myo-Inositol 1-phosphate**	1,9453	0,50021
**Mannose 6-phosphate**	1,9453	0,50021
**Pyruvic acid**	2,0247	0,22619
**Sphinganine**	2,0614	0,15472
**L-Fucose**	2,1266	0,17253
**Rhamnose**	2,1266	0,17253
**Palmitoylethanolamide**	2,1461	0,18723
**Imidazolepropionic acid**	2,155	0,1654
**Sphingosine**	2,168	0,18912
**3-Dehydrosphinganine**	2,168	0,18912
**Indolelactic acid**	2,3216	0,52521
**Indoleacrylic acid**	2,3276	0,52607
**4-Hydroxycyclohexylcarboxylic acid**	2,341	0,19461
**Suberic acid**	2,5041	0,19157
**Cysteine-S-sulfate**	2,54	0,20727
**L-Tryptophan**	2,5922	0,20304
**L-Proline**	2,6795	0,20849
**Heptadecanoic acid**	2,7756	0,21065
**Tyramine**	2,7811	0,39607
**Myristoleic acid**	2,84	0,21925
**Azelaic acid**	2,8611	0,22474
**Methylglutaric acid**	3,2228	0,2997
**2-Methylglutaric acid**	3,2228	0,2997
**Adipic acid**	3,2228	0,2997
**Stearic acid**	3,2899	0,31457
**N-Acetyl-L-phenylalanine**	3,3722	0,34308
**Phenylpropionylglycine**	3,3722	0,34308
**trans-4-Hydroxycyclohexylacetic acid**	3,3912	0,32551
**cis-4-Hydroxycyclohexylacetic acid**	3,3912	0,32551
**N-Acetylcadaverine**	3,4851	0,3244
**Prostaglandin F2a**	3,6412	0,33119
**Ergosterol**	3,6435	0,25737
**3-Hydroxyoctanoic acid**	3,6824	0,34329

**7-Hydroxyoctanoic acid**	3,6824	0,34329
**R-2-Hydroxycaprylic acid**	3,6824	0,34329
**Hydroxyoctanoic acid**	3,6824	0,34329
**4-Trimethylammoniobutanoic acid**	3,7402	0,52509
**Mevalonic acid**	3,7433	0,34007
**2-Furoylglycine**	3,9775	0,39841
**3-Methylthiopropionic acid**	4,0497	0,36138
**Traumatic acid**	5,1547	0,41578
**Cholesterol**	5,4002	0,4873
**Niacinamide**	5,4451	0,45016
**Aminomalonic acid**	5,8664	0,56411
**3b-Hydroxy-5-cholenoic acid**	5,9675	0,70329
**Isoursodeoxycholic acid**	6,7184	0,55999
**Isodeoxycholic acid**	6,7184	0,55999
**Deoxycholic acid**	6,7184	0,55999
**Chenodeoxycholic acid**	6,7184	0,55999
**Ursodeoxycholic acid**	6,7184	0,55999
**2-Oxo-4-methylthiobutanoic**	6,9855	0,55572
**Dodecanedioic acid**	7,1703	0,55102
**8-Hydroxyguanine**	7,6075	0,64709
**L-Lysine**	7,7921	0,64855
**PC1819Z/1819Z**	7,8345	0,83144
**alpha-Tocopherol**	8,0793	0,66417

Table 3 . Markers S-plot comparing CFS patients vs controls.

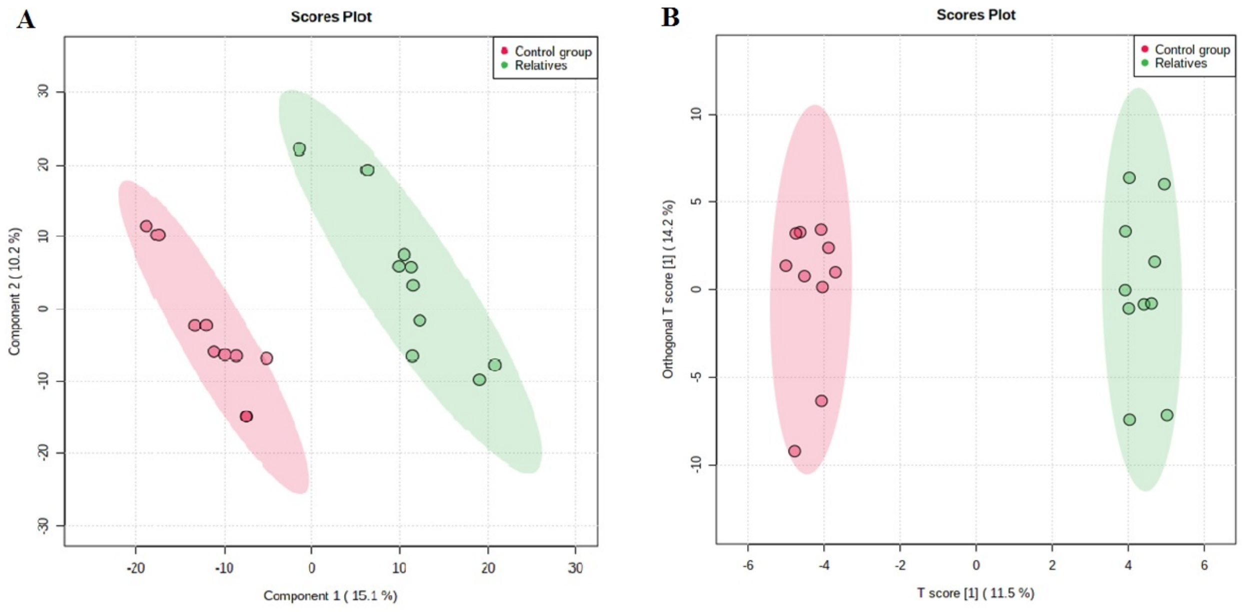

Figure 11. (A) PLS-DA and (B) OPLS-DA supervised models for the classification of the experimental groups (relatives vs controls). In the score plot of OPLS-DA, the x-axis represents the biological component and the y-axis the orthogonal component.

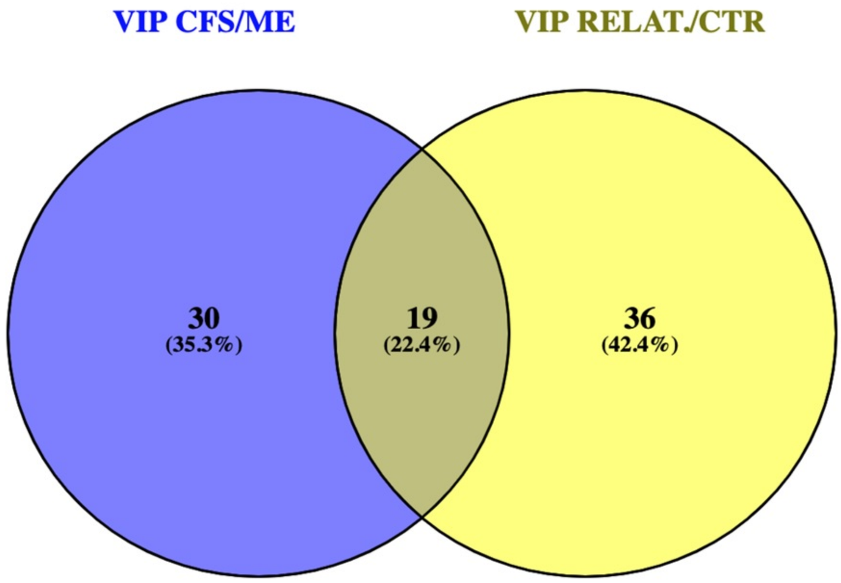

Figure 12. Venn diagram. Discriminant metabolites between the three experimental groups based on VIP scores.
